# CSF rhinorrhoea after endonasal intervention to the skull base (CRANIAL): A multicentre prospective observational study

**DOI:** 10.3389/fonc.2022.1049627

**Published:** 2023-01-04

**Authors:** CRANIAL Consortium, Danyal Z Khan

**Keywords:** cerebrospinal fluid rhinorrhoea, CSF, EEA, endoscopic endonasal, cerebrospinal fluid leak, skull base surgery

## Abstract

**Objective:**

Despite progress in endonasal skull-base neurosurgery, cerebrospinal fluid (CSF) rhinorrhoea remains common and significant. The CRANIAL study sought to determine 1) the scope of skull-base repair methods used, and 2) corresponding rates of postoperative CSF rhinorrhoea in the endonasal transsphenoidal approach (TSA) and the expanded endonasal approach (EEA) for skull-base tumors.

**Methods:**

A prospective observational cohort study of 30 centres performing endonasal skull-base neurosurgery in the UK and Ireland (representing 91% of adult units). Patients were identified for 6 months and followed up for 6 months. Data collection and analysis was guided by our published protocol and pilot studies. Descriptive statistics, univariate and multivariable logistic regression models were used for analysis.

**Results:**

A total of 866 patients were included - 726 TSA (84%) and 140 EEA (16%). There was significant heterogeneity in repair protocols across centres. In TSA cases, nasal packing (519/726, 72%), tissue glues (474/726, 65%) and hemostatic agents (439/726, 61%) were the most common skull base repair techniques. Comparatively, pedicled flaps (90/140, 64%), CSF diversion (38/140, 27%), buttresses (17/140, 12%) and gasket sealing (11/140, 9%) were more commonly used in EEA cases. CSF rhinorrhoea (biochemically confirmed or requiring re-operation) occurred in 3.9% of TSA (28/726) and 7.1% of EEA (10/140) cases. A significant number of patients with CSF rhinorrhoea (15/38, 39%) occurred when no intraoperative CSF leak was reported. On multivariate analysis, there may be marginal benefits with using tissue glues in TSA (OR: 0.2, CI: 0.1-0.7, p<0.01), but no other technique reached significance. There was evidence that certain characteristics make CSF rhinorrhoea more likely – such as previous endonasal surgery and the presence of intraoperative CSF leak.

**Conclusions:**

There is a wide range of skull base repair techniques used across centres. Overall, CSF rhinorrhoea rates across the UK and Ireland are lower than generally reported in the literature. A large proportion of postoperative leaks occurred in the context of occult intraoperative CSF leaks, and decisions for universal sellar repairs should consider the risks and cost-effectiveness of repair strategies. Future work could include longer-term, higher-volume studies, such as a registry; and high-quality interventional studies.

## Introduction

Endonasal approaches have revolutionized skull-base neurosurgery (1, 2). The most commonly utilized approach is the transsphenoidal approach (TSA), frequently used for sellar lesions. More recently, the development of the expanded endonasal approach (EEA) has allowed access to pathologies extending beyond the sella, with growing indications as this technique evolves (3, 4).

An international expert consensus on TSA workflow highlighted the potential for practice variations, particularly in closure, due to a variety of skull-base repair options (5). Previous systematic reviews examining skull-base repair techniques across endonasal skull-base neurosurgery found absolute heterogeneity across studies and centres, likely due to a paucity of high-level comparative evidence (6). Similarly, there is variance in postoperative cerebrospinal fluid (CSF) rhinorrhoea rates, one of the commonest postoperative complications – generally up to 5% in TSA and 20% in EEA (4, 7–12). CSF rhinorrhoea has potentially serious consequences including pneumocephalus, meningitis, and prolonged hospital admission or re-admission (9, 13, 14).

CRANIAL (CSF Rhinorrhoea After Endonasal Intervention to the Skull Base) was a prospective, multicentre observational study seeking to determine the: (1) scope of the methods of skull-base repair; and (2) corresponding rates of postoperative CSF rhinorrhoea in the UK and Ireland (15–17). CRANIAL was a collaboration between three bodies: students and junior doctors *via* NANSIG (The Neurology and Neurosurgery Interest Group), neurosurgical trainees *via* BNTRC (British Neurosurgical Trainee Research Collaborative) and skull-base consultants (neurosurgery and otorhinolaryngology) *via* the CRANIAL Steering Committee.

After piloting at 12 centres, preliminary results suggested practice heterogeneity (15, 16). Thus, the study was expanded UK and Ireland wide, and herein, we present the results.

## Methods

The Strengthening the Reporting of Observational Studies in Epidemiology (STROBE) statement guided this methodology and report (18).

### Study design

A multicentre, prospective, observational cohort study design was conducted across tertiary neurosurgical units with 2 pilot phases (Phase 1, 4 centres, 01/11/2019-22/03/2020; Phase 2, 12 centres, 23/03/2020-31/07/2020) and a full study period (15–17). The full study included 30 centres, representing 91% (29/32, of adult neurosurgical centres performing endonasal skull-base neurosurgery in the UK and Ireland). One pediatric centre was included, whilst others provided both adult and pediatric services. The study period included 6 months of consecutive case recruitment (10/08/20–10/02/21) and 6 months of follow-up (10/02/21–10/08/21) (19).

Cases included patients of all ages undergoing TSA for sellar tumors and EEA for skull base tumors (17). TSA was defined as surgical access to the sella alone (transsphenoidal) whilst EEA was defined as acquiring surgical access to an area not limited to the sella (e.g., transplanum or transcribriform) (17, 20). Exclusion criteria were patients undergoing transcranial surgery and those with preoperative CSF rhinorrhoea.

### Data collection

Each centre registered the project as a service evaluation with appropriate approvals. Following the BNTRC model (21), the local team consisted of consultant lead(s) with overall project responsibility, with trainee lead(s) and student lead(s) for data collection *via* a secure web-based central database (Castor Electronic Data Capture). NANSIG and the BNTRC provided project support, overseen by the CRANIAL consultant steering committee.

Data were collected as per protocol (15–17). The Esposito-Kelly system graded intraoperative CSF leak if present (22). Local teams aimed to collect data within 30 days of operation for admission data, and at the end of the 6-month follow-up window for follow-up data (17). Primary outcomes were: (1) methods of intraoperative skull-base reconstruction, and (2) postoperative CSF rhinorrhoea biochemically confirmed or requiring intervention (CSF diversion and/or operative repair) (17).

### Data validation

Data were confirmed with operating surgeons or senior team members before final submission. An independent local data validator screened a random 10% of submitted cases at each centre. The primary validation target was >95% accuracy across audited data (17). Finally, each local team reviewed their final validated dataset before analysis.

### Data analysis

Pre-processing included re-categorizing free-text entries. Descriptive statistics summarized baseline characteristics (demographic, tumour, and operative characteristics) and surgical outcomes, using Microsoft Excel (Version 16.54). The incidence density of repair methods and combinations within TSA/EEA and CSF leak grade subgroups were calculated. Corresponding postoperative CSF rhinorrhoea rates were summarized as incidence percentages per TSA/EEA subgroups and repair method used. Univariate and multivariable logistic regression models assessed the impact of baseline characteristics (from the literature) on skull-base repair methods, and the influence of baseline characteristics and skull-base repair methods on CSF rhinorrhoea incidence, with odds ratios and 95% confidence intervals reported (Stata, Version 16, StataCorp, USA) (17). Fisher’s exact test was used to compare repair methods used with and without intraoperative CSF leak.

## Results

866 patients (726 TSA, 140 EEA) were included across 30 centres. All centres completed data validation, with >95% record accuracy in audited cases and no duplicates included.

### Patient characteristics

The median patient age was 53 years (range: 5–84), 23% (198/866) were older than 65. There were 416 male patients and 450 female patients; 238 (TSA: 210/726; EEA: 28/140) patients were obese (body mass index >30) (**Tables 1**, **2**). Pre-operative visual deficits (acuity and/or field) were present in 464 patients (TSA: 374/726; EEA: 91/140); 6 were blind with binocular <6/60 acuity (TSA: 9/374; EEA: 3/91) (**Supplementary Material 6**). Pre-operative anterior hypopituitarism (requiring hydrocortisone supplementation) was present in 215 cases (TSA: 184/726; EEA: 31/140), and posterior hypopituitarism (requiring desmopressin supplementation) in 36 cases (TSA: 28/726; EEA: 8/140). The commonest TSA pathologies were non-functioning pituitary adenoma (410/726), functioning pituitary adenoma (249/726), and Rathke’s cleft cyst (26/726) (**Supplementary Material 3**). For EEA, craniopharyngioma (38/140), meningioma (25/140) and non-functioning pituitary adenoma (23/140) were the commonest. Most tumors were >1cm in maximum diameter (TSA: 607/726; EEA: 131/140).

**Table 1:** Incidence of repair technique categories across surgical approaches, intraoperative CSF leak presence/grade, tumour diameter, BMI and age.

Category	Dural closure	Dural replacement	Tissue graft	Synthetic graft	Button technique	Pedicled flap	Tissue glue	Haemostatic agent	Buttress	Gasket sealing	Nasal packing	CSF diversion	CSF rhinorrhoea
**Approach**													
TSA (N = 726), n (n/N%)	0 (0%)	196 (27%)	221 (30.4%)	204 (28.1%)	20 (2.8%)	116 (16%)	474 (65.3%)	439 (60.5%)	31 (4.3%)	15 (2.1%)	519 (71.5%)	29 (4%)	28 (3.9%)
EEA (N = 140), n (n/N%)	0 (0%)	66 (47.1%)	65 (46.4%)	47 (33.6%)	7 (5%)	90 (64.3%)	114 (81.4%)	93 (66.4%)	17 (12.1%)	11 (7.9%)	116 (82.9%)	38 (27.1%)	10 (7.1%)
**Intraoperative CSF leak grade**													
Grade 0 (N = 573), n (n/N%)	0 (0%)	136 (23.7%)	106 (18.5%)	163 (28.4%)	9 (1.6%)	88 (15.4%)	335 (58.5%)	358 (62.5%)	19 (3.3%)	11 (1.9%)	403 (70.3%)	19 (3.3%)	15 (2.6%)
Grade 1 (N = 143), n (n/N%)	0 (0%)	54 (37.8%)	89 (62.2%)	45 (31.5%)	7 (4.9%)	37 (25.9%)	124 (86.7%)	82 (57.3%)	7 (4.9%)	3 (2.1%)	114 (79.7%)	13 (9.1%)	4 (2.8%)
Grade 2 (N = 67), n (n/N%)	0 (0%)	27 (40.3%)	41 (61.2%)	18 (26.9%)	7 (10.4%)	33 (49.3%)	55 (82.1%)	33 (49.3%)	10 (14.9%)	4 (6%)	52 (77.6%)	8 (11.9%)	10 (14.9%)
Grade 3 (N = 44), n (n/N%)	0 (0%)	23 (52.3%)	33 (75%)	15 (34.1%)	3 (6.8%)	30 (68.2%)	44 (100%)	28 (63.6%)	9 (20.5%)	6 (13.6%)	31 (70.5%)	16 (36.4%)	2 (4.5%)
Grade unknown (N = 39), n (n/N%)	0 (0%)	22 (56.4%)	17 (43.6%)	10 (25.6%)	1 (2.6%)	18 (46.2%)	30 (76.9%)	31 (79.5%)	1 (2.6%)	2 (5.1%)	18 (46.2%)	46.2 (30%)	7 (17.9%)
**Specialty**													
Neurosurgery only (N=505), n (n/N%)	0 (0%)	154 (30.5%)	219 (43.4%)	164 (32.5%)	24 (4.8%)	63 (12.5%)	361 (71.5%)	274 (54.3%)	33 (6.5%)	21 (4.2%)	297 (58.8%)	40 (7.9%)	21 (4.2%)
Otorhinolaryngology only (N=25), n (n/N%)	0 (0%)	17 (68%)	2 (8%)	14 (56%)	0 (0%)	5 (20%)	25 (100%)	25 (100%)	0 (0%)	0 (0%)	25 (100%)	0 (0%)	4 (16%)
Multidisciplinary (N=336), n (n/N%)	0 (0%)	91 (27.1%)	65 (19.3%)	73 (21.7%)	3 (0.9%)	138 (41.1%)	202 (60.1%)	233 (69.3%)	15 (4.5%)	5 (1.5%)	313 (93.2%)	27 (8%)	13 (3.9%)
**Tumour diameter**													
>1cm (N=738), n (n/N%)	0 (0%)	238 (32.2%)	243 (32.9%)	218 (29.5%)	26 (3.5%)	190 (25.7%)	510 (69.1%)	456 (61.8%)	44 (6%)	24 (3.3%)	546 (74%)	61 (8.3%)	31 (4.2%)
<1cm (N=128), n (n/N%)	0 (0%)	24 (18.8%)	43 (33.6%)	33 (25.8%)	1 (0.8%)	16 (12.5%)	78 (60.9%)	76 (59.4%)	4 (3.1%)	2 (1.6%)	89 (69.5%)	6 (4.7%)	7 (5.5%)
**BMI**													
<30 (N=628), n (n/N%)	0 (0%)	190 (30.3%)	211 (33.6%)	181 (28.8%)	20 (3.2%)	148 (23.6%)	416 (66.2%)	378 (60.2%)	41 (6.5%)	24 (3.8%)	456 (72.6%)	51 (8.1%)	25 (4%)
>30 (N=238), n (n/N%)	0 (0%)	72 (30.3%)	75 (31.5%)	70 (29.4%)	7 (2.9%)	58 (24.4%)	172 (72.3%)	154 (64.7%)	7 (2.9%)	2 (0.8%)	179 (75.2%)	16 (6.7%)	13 (5.5%)
**Age**													
<65 (N=668), n (n/N%)	0 (0%)	201 (30.1%)	216 (32.3%)	197 (29.5%)	19 (2.8%)	168 (25.1%)	462 (69.2%)	419 (62.7%)	35 (5.2%)	17 (2.5%)	493 (73.8%)	54 (8.1%)	35 (5.2%)
>65 (N=198), n (n/N%)	0 (0%)	61 (30.8%)	70 (35.4%)	54 (27.3%)	8 (4%)	38 (19.2%)	126 (63.6%)	113 (57.1%)	13 (6.6%)	9 (4.5%)	142 (71.7%)	13 (6.6%)	3 (1.5%)

CSF, cerebrospinal fluid; BMI, body mass index.

**Table 2:** Summary of CSF rhinorrhoea incidence per baseline and operative risk factor subgroups – incidence and statistical analysis *via* multivariate logistic regression.

	*Transsphenoidal approach*		*Expanded Endonasal Approach*	
	CSF Rhinorrhoea rate	Multivariate Analyses (OR, CI, p-value)	CSF Rhinorrhoea rate	Multivariate Analyses (OR, CI, p-value)
*Approach*				
*TSA*	28/726 (3.9%)	–	–	–
*EEA*	–	–	10/140 (7.1%)	–
*Baseline characteristics*				
*Age >65*	0/172 (0.0%)	–	3/27 (11.1%)	OR: 3.8, CI: 0.6–23.7, p =0.16
*Age <65*	28/553 (5.1%)	Reference	7/113 (6.2%)	Reference
*BMI >30*	11/210 (5.2%)	OR: 1.7, CI: 0.7-4.4, p=0.26	2/28 (7.1%)	OR: 0.7, CI: 0.1-6.1, p=0.7
*BMI<30*	17/516 (3.3%)	Reference	8/112 (7.1%)	Reference
*Tumour diameter >1cm*	21/607 (3.5%)	OR:0.5; CI: 0.2 – 1.5, p=0.22	10/131 (7.6%)	–
*Tumour diameter <1cm*	7/119 (6.0%)	Reference	0/9 (0%)	Reference
*Primary surgery*	8/98 (8.2%)	OR:0.4, CI: 0.1-0.9, p=0.05	1/21 (4.8%)	OR: 0.6, CI; 0.1-8.4, p=0.71
*Revision surgery*	19/573 (3.3%)	Reference	7/113 (6.2%)	Reference
*Presence of Otorhinolaryngologist*	9/268 (3.4%)	OR: 0.4, CI: 0.1-1.6, p=0.2	8/93 (8.6%)	OR: 0.6, CI: 0.1-7.4, p=0.72
*Presence of Neurosurgeon*	25/704 (3.6%)	OR: 0.2, CI: 0.1-1.9, p=0.17	9/137 (6.6%)	OR: 0.1, CI: 0-1.8, p=0.1
*Intra-operative CSF leak grade*				
*Grade 0*	11/512 (2.1%)	Reference	4/61 (6.6%)	Reference
*Grade 1*	3/131 (2.3%)	OR: 1.5, CI: 0.4-6.6, p=0.56	1/12 (8.3%)	OR: 2.2, CI: 0.1-39.9, p= 0.61
*Grade 2*	9/54 (16.7%)	OR: 16.1, CI: 4.6-56.3, p<0.01	1/13 (7.7%)	OR: 1.8, CI: 0.1-24.2, p=0.67
*Grade 3*	0/5 (0%)	-	2/39 (5.6%)	OR: 1.2, CI: 0.1-11.5, p=0.87
*Leak present, grade unknown*	5/24 (20.8%)	OR: 7.6, CI: 1.8-33.4, p<0.01	2/15 (13.3%)	OR: 12, CI: 0.4-356.3, p=0.15
*Repair methods*				
*Dural closure*	–	–	–	–
*Dural replacement*	11/196 (5.6%)	OR: 2.6, CI: 0.8-8.8, p=0.13	5/66 (7.6%)	OR: 0.9, CI: 0.1-5.1, p=0.85
*Tissue graft*	13/221 (5.9%)	OR: 1.8, CI: 0.6-5.3, p=0.29	3/65 (4.6%)	OR: 0.3, CI: 0.1-2.2, p=0.21
*Synthetic graft*	7/204 (3.4%)	OR: 1.2, CI: 0.4-3.6, p=0.79	6/47 (12.8%)	OR: 5.2, CI: 0.7-39.1, p=0.11
*Button Technique*	0/20 (0%)	–	0/7 (0%)	–
*Pedicled Flap*	5/116 (4.3%)	OR: 0.9, CI: 0.3-3.2, p=0.87	8/90 (8.9%)	–
*Tissue Glue*	15/474 (3.2%)	OR: 0.2, CI: 0.1-0.7, p<0.01	8/114 (7.0%)	OR: 4.4, CI: 0.3-78.6, p=0.31
*Haemostatic agent*	18/439 (4.1%)	OR: 1.3, CI: 0.5-3.4, p=0.63	5/93 (5.4%)	OR: 0.3, CI: 0.1-2.5, p=0.27
*Buttress*	0/31 (0%)	–	1/17 (5.9%)	OR: 2.8, CI: 0.1-63.1, p=0.53
*Gasket sealing*	0/15 (0%)	–	0/11 (0%)	–
*Nasal packing*	22/519 (4.2%)	OR: 1.9, CI: 0.6-5.8, p=0.29	10/116 (8.6%)	–
*CSF diversion*	1/29 (3.4%)	OR: 0.9, CI: 0.1-8.3, p=0.96	1/38 (2.6%)	OR: 0.2, CI: 0-5.3, p =0.298

### Operation characteristics

Of TSA cases, endoscopic was most prevalent (615/726), followed by microscopic (80/726), and a combined approach (32/726) method. Revision surgery was infrequent (TSA 98/726; EEA 21/140). On multivariate logistic regression, TSA was less likely to be used for larger tumors (maximum diameter >1cm) compared to EEA, aligning with indications for these approaches (OR: 0.4, CI: 0.2-0.9, p=0.03). Most TSA surgeries were performed by neurosurgeons alone (458/726), whereas most EEA cases were performed with both neurosurgery and otorhinolaryngology specialists (90/140). Infrequently cases were performed by otorhinolaryngologists alone (TSA: 22/726; EEA: 3/140). The median operation duration was 110 minutes for TSA (range: 29–540 minutes) and 220 minutes for EEA (range: 30–795 minutes).

Intraoperative CSF leak was reported in 214 TSA cases (214/726) and 79 EEA cases (79/140). Intraoperative CSF leaks were most commonly low-flow in TSA (131/214 grade 1) and high-flow in EEA (39/79 grade 3) (**Tables 1**, **2**).

### Skull-base reconstruction overview

**Figure 1:**
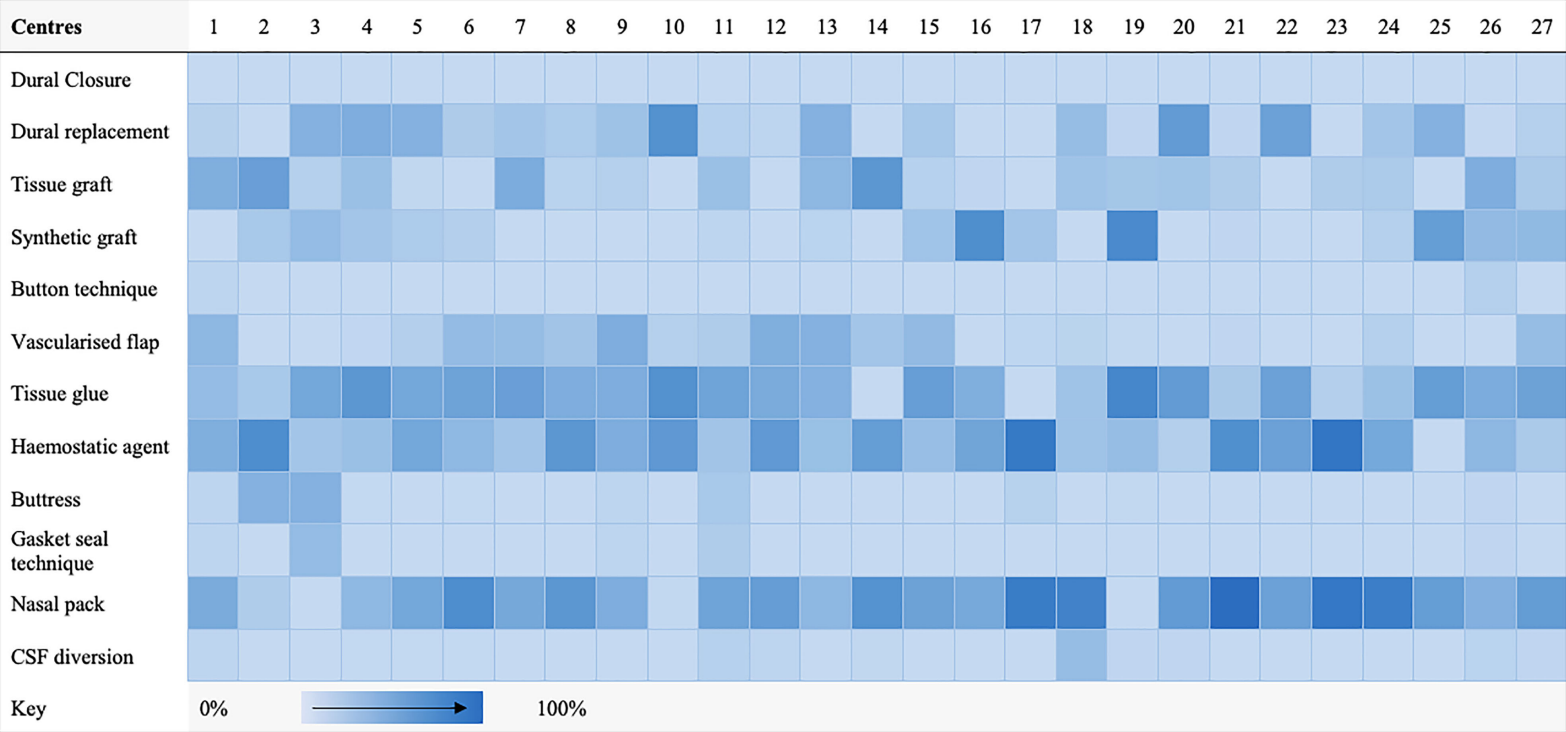
Heat map highlighting frequency of repair technique category use per centre for transsphenoidal cases.

**Figure 2:**
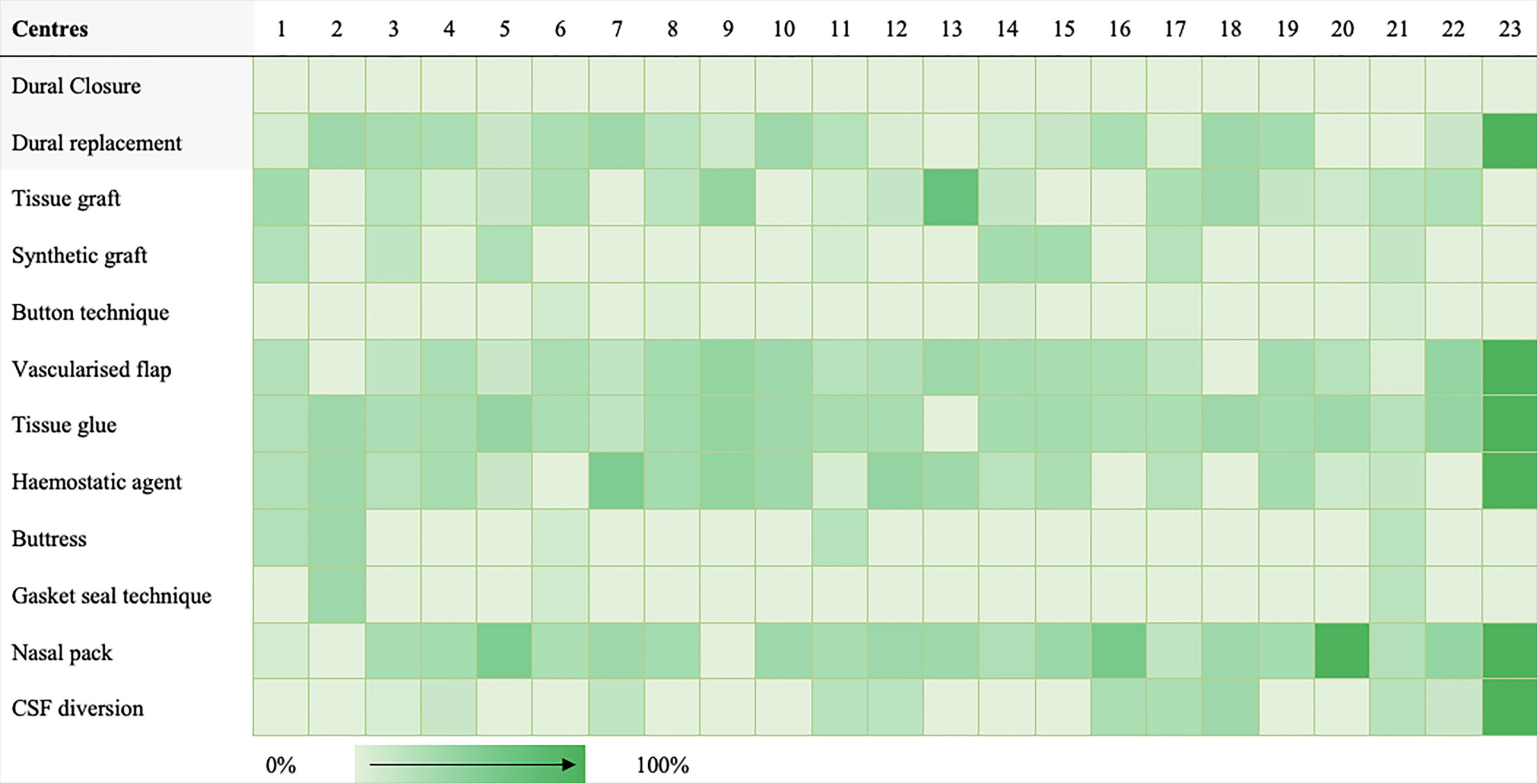
Heat map highlighting frequency of repair technique category use per centre for expanded endonasal cases.

A taxonomy for skull-base repair was adapted from a systematic review of the literature (**Supplementary Material 2**) (20, 21). Heterogeneity of repair technique choice across both approaches was evident (**Figures 1**, **2**).

In TSA, the commonest techniques were nasal packing (519/726), tissue glues (474/726) and hemostatic agents (439/726) (**Table 1**; **Supplementary Material 4**). The most prevalent nasal packing was Nasopore^®^ (369/519), Merocel^®^ (94/519) and Rapid Rhinos^®^ (33/519). Tissue glues most frequently used were Adherus^®^ (146/474), Duraseal^®^ (137/474) and Tisseel^®^ (126/474); whilst common hemostatic agents included Surgicel^®^ (189/439), Surgiflo^®^ (141/439) and Floseal^®^ (91/439). Tissue grafts were used in 221 cases (221/726), usually fat (189/221, most commonly abdominal), fascia (27/221, most often fascia lata) and mucosa (28/221, usually middle turbinate). Synthetic grafts (204/726) included Spongostan™ (181/204), Tachosil^®^ (21/204) and Gelfoam^®^ (2/204). The button technique was used with these grafts in 20 cases (20/726). There was overlap between these graft materials and dural replacement (or reconstruction *via* layering) strategies (196/726) which usually consisted of Duragen^®^ (136/196), fascia lata (18/196) or Lyoplant^®^ (17/196). Pedicled flaps were used in 116 cases (116/726), most frequently nasoseptal flaps (105/116). Rigid buttresses were used in 31 cases (31/726), commonly Medpor^®^ (15/31), autologous bone (14/31, usually septal) and autologous cartilage (1/31). These buttresses were used with a gasket seal technique in 15 cases (15/726), usually with fascia lata.

Comparatively, pedicled flaps (90/140), CSF diversion (38/140), buttresses (17/140), and gasket sealing (11/140) were more commonly used in EEA cases (**Table 1**; **Supplementary Material 4**). Nasoseptal flaps (87/90) were again the most frequent pedicled flaps. Like TSA, supportive buttresses were often Medpor^®^ (10/17) or autologous bone (5/17), the majority of these being used with the gasket seal technique (11/17). Additionally, nasal packs (116/140), tissue glue (114/140) and hemostatic agents (93/140) were prevalent. The commonest nasal packs were Nasopore^®^ (86/116), Merocel^®^ (20/116) and Bismuth-Soaked Ribbon Gauze (11/116). Again, Tisseel^®^ (32/114), Adherus^®^ (22/114) and Duraseal^®^ (22/114) were the most used tissue glues; whilst Surgicel^®^ (51/93), Surgiflo^®^ (24/93) and Floseal^®^ (13/93) were common hemostatic agents. Tissue grafts (65/140), were frequently fat (45/65), fascia (36/65) and mucosa (8/65), akin to TSA. Similarly, synthetic grafts (47/140) included Spongostan™ (39/47) and Tachosil^®^ (5/47). The button technique was sometimes used with these grafts (47/140). Finally, common dural replacement (66/140) strategies included Duragen^®^ (43/66), fascia lata (12/66) and Tutoplast^®^ (6/66).

### Factors affecting repair technique choice

Repair methods appeared to be tailored according to postoperative CSF rhinorrhoea risk (**Table 1** for descriptive analyses, **Supplementary Material 5** for further statistical analyses). In cases with intraoperative CSF leak, there was a statistically significant (via Fisher’s exact test) increased use of tissue grafts (p<0.01), pedicled flaps (p<0.01), tissue glues (p<0.01) and CSF diversion (TSA p<0.01; EEA p<0.05) for both TSA and EEA on univariate analysis. Additionally, dural replacements (p<0.01), hemostatic agents (p=0.01) and buttresses (p<0.01) were also used more in EEA (but not TSA) with intraoperative CSF leak. Similarly, the use of pedicled flaps (OR: 2.3, CI: 1.3-4.2, p=0.01), dural replacement (OR: 2.1, CI: 1.3-3.4, p<0.01) and tissue glues (OR: 1.36, CI: 1.1-2.5, p=0.02) were statistically associated with operations for larger tumors (maximum diameter >1cm) on multivariate logistic regression. Regarding surgical specialty, the use of pedicled flaps (OR: 4.5, CI: 3.1-6.3, p<0.01) and hemostatic agents (OR: 1.9, CI: 1.5-2.7, p<0.01) were statistically associated with otorhinolaryngology involvement, whilst the use of tissue grafts (OR: 0.3, CI: 0.2-0.5, p<0.01) and tissue glues (OR: 0.6, CI: 0.4-0.8, p<0.01) was reduced on multivariate logistic regression.

### CSF diversion

67 cases used CSF diversion (TSA: 29/726; EEA: 38/140). In TSA, lumbar drainage was most common (27/29) with one of these patients subsequently requiring a ventriculoperitoneal shunt (VPS). The remainder underwent lumbar puncture (1/29), or external ventricular drain (EVD) placement (1/29). Lumbar drains were usually placed under the same anesthetic (pre-procedure, 15/29; post-procedure, 7/29), with regimes (if specified) volume-led (14/29, usually 5-10mls/hr), pressure-led (6/29) or using a LiquoGuard^®^ system (1/29). Three drains inserted pre-procedure were removed before any effective postoperative CSF drainage (used for intraoperative saline injection or inserted prophylactically in case of subsequent CSF rhinorrhoea). Excluding these, the median length of drainage *via* lumbar drain was five days (range: 2-11).

Regarding EEA surgeries, all CSF diversion was performed *via* lumbar drain with most placed under the same anesthetic (immediately pre-procedure: 22/38; or immediately post-procedure: 8/38). The most common drainage regime was volume-led (21/22), with 5-10mls/hr the commonest protocol. One case also had an EVD placed one week before tumour resection for acute hydrocephalus. Three pre-procedure drains inserted were removed before any effective postoperative CSF drainage. Excluding these, the median length of drainage was five days (range: 1-7).

### Postoperative management

The median patient hospital stay was four days (range: 1–37) for TSA and seven days (range: 1–35) for EEA. Regarding conservative measures, bed rest was advised in 20% (147/726) TSA cases (head elevated: 72/147; head flat: 5/147; unspecified height: 70/152) and 40% (52/140) EEA cases (head elevated: 37/52; head flat: 3/52; unspecified height: 12/52). Avoiding straining (e.g., lifting, sneezing, etc.) was advised in most TSA (502/726) and EEA (91/140) cases. Stool softeners were prescribed in 191 TSA cases (191/726) and 30 EEA cases (30/140). Rarely, acetazolamide (TSA: 1/726; EEA 1/140) was offered. Visual outcomes, endocrine outcomes and complications at 6 months follow-up are summarized in **Supplementary Material 6**.

### Postoperative CSF rhinorrhoea

CSF rhinorrhoea (biochemically confirmed or requiring re-operation) occurred in 3.9% of TSA (28/726) and 7.1% of EEA (10/140) cases.

In TSA, most cases occurred during the index admission (21/28), presenting a median of 2 days postoperatively (range: 1-17), whereas those presenting during follow-up (7/28) a median of 10 days postoperatively (range: 2-84). Almost all cases were managed operatively (index: 18/21; follow-up: 6/7). Initial surgical treatment included lumbar drain alone (8/24), lumbar drain & direct endonasal repair (8/24), direct endonasal repair alone (6/24), or VPS alone (2/24). Five cases required further operations for recurrent CSF rhinorrhoea. Regarding EEA, CSF rhinorrhoea occurred during the index admission for 8 cases, and during follow-up for 2 cases. All cases were managed operatively (lumbar drain & endonasal repair: 6/10; lumbar drain alone 3/10; endonasal repair alone: 1/10). Two cases required further operations for recurrent CSF rhinorrhoea. Cases presenting during index admission were detected at a median of 2 days postoperatively (range: 1-11), whilst those detected during follow-up were found at a median of 19 days postoperatively (range: 8-54).

**Figure 3:**
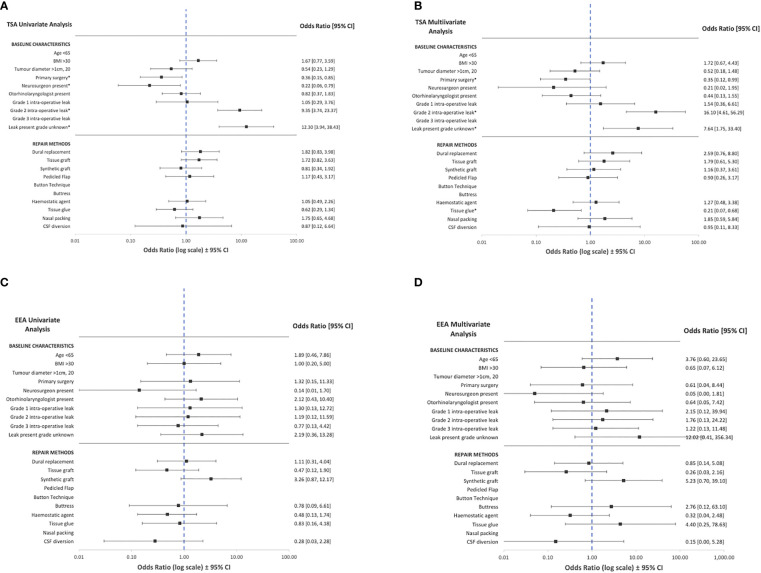
Summary of univariate and multivariate logistic regression of baseline characteristics and operative technique against CSF rhinorrhoea across transsphenoidal **(A, B)** and expanded endonasal **(C, D)** appraoches. CSF, cerebrospinal fluid, BMI=body mass index, TSA=transsphenoidal approach; EEA, expanded endonasal approach. *Statistically significant relationships (p<0.05, see **Table 2** and **Supplementary Information 3**).

On univariate logistic regression analysis, displayed in **Figure 3**, the following variables were associated with CSF rhinorrhoea: revision surgery (TSA), presence of intraoperative CSF leak (TSA), and the absence of neurosurgery involvement (TSA) (**Table 2**; **Figure 3**; **Supplementary Material 5**). On multivariate analysis, revision surgery and the presence of intraoperative CSF leak remained a predictor of CSF rhinorrhoea in TSA (**Table 2**; **Figure 3**; **Supplementary Material 5**). No specific technique category (including CSF diversion) considerably impacted the odds of CSF rhinorrhoea for EEA. However, tissue glues in TSA (OR: 0.2, CI: 0.1-0.7, p<0.01) may be related to a slight decrease in CSF rhinorrhoea rates on multivariate analyses (**Table 2**; **Figure 3**; **Supplementary Material 5**).

## Discussion

### Principal findings

This multicentre, prospective, observational study represents the first study of its kind, exploring skull base repair techniques and CSF rhinorrhoea rates in a collaborative project involving almost all neurosurgical centres in the UK and Ireland.

There is clear heterogeneity in skull-base repair regimes across centres, with no two sharing the same protocol. Additionally, no specific type of repair technique made a significant difference in postoperative CSF rates, although there may be marginal benefits with tissue glue in TSA. Certain characteristics appear to make CSF rhinorrhoea more likely – previous endonasal surgery and intraoperative CSF leak. This translates into the tailoring of repair strategies. For example, in EEA, multilayer regimes using pedicled flaps, rigid buttresses (often with gasket sealing) and CSF diversion were frequent. Similarly, in the context of intraoperative CSF leak, tissue grafts, tissue glues, pedicled flaps and CSF diversion were used more often. Larger tumors (maximum diameter >1cm) were associated with the use of pedicled flaps, dural replacement and tissue glues. Surgeon preference or training may also factor in, with pedicled flaps and hemostatic agents used less in the absence of otorhinolaryngologists. Tissue grafts, tissue glues, and construct support strategies (e.g., rigid buttresses and CSF diversion) were less frequent in the absence of neurosurgical involvement.

CSF rhinorrhoea for both TSA (28/726, 3.9%) and EEA (10/140, 7.1%) are lower than generally reported in the literature (6, 7, 9, 10, 12, 23). This may reflect the ongoing improvement in endonasal skull-base repair and CSF rhinorrhoea rates, demonstrated by recent meta-analyses over time (24). Additionally, the UK and Ireland are consolidating pituitary services into dedicated “centres of excellence”, which may influence surgical outcomes (25). Furthermore, as a prospective series, surgeons were aware of the monitoring of this outcome, perhaps influencing their management *via* the *Hawthorne effect (* 26). Importantly, a significant proportion of postoperative CSF rhinorrhoea cases had no recorded intraoperative CSF leak (Total: 15/38; TSA: 11/28; EEA: 4/10), suggesting occult intraoperative leak, or possibly a thinned and vulnerable arachnoid dome which tears postoperatively in the absence of support. In our series, this subgroup had the lowest frequency of almost every repair method category (except synthetic grafts and hemostatic agents). This phenomenon is described in other case series, with many authors advocating for universal sellar repair for this reason, and some recommending routine use of intrathecal fluorescein (27, 28). However, these strategies should be balanced against the increased operative time, cost-effectiveness, and additional repair-related morbidity (e.g., donor site injuries or scars) (27, 28).

### Findings in the context of literature

The development of endonasal techniques have revolutionized skull base surgery by allowing direct access to the skull base regions *via* a natural working channel which although long and narrow, accommodates specialized long instruments (1, 29–31). Gravity and the pressure gradient across the surgical bony/dural opening often assists surgical resection, for example in the descent of softer pituitary tumours, and is sometimes manipulated through pressure modulation (e.g., Valsalva and intrathecal saline injection) (1, 29–31). However, these advantages also contribute to the endonasal approach’s inherent susceptibility of CSF rhinorrhoea – repairing the skull base using long rigid instruments *via* a narrow surgical corridor, with restricted motion and dexterity, against gravity and CSF pressure, creating a significant surgical challenge (1, 6, 29–31). This challenge has been met by refinements in endoscopic and microscope techniques, however, large variations in CSF rhinorrhoea rates still exist in both transsphenoidal and expanded endonasal surgery (6, 24, 32). An important component of this refinement has been the development of new closure strategies (24, 33–35).

However, recent systematic reviews of skull-base repair techniques have highlighted the variations across surgeons and centres, likely related to the lack of high-level comparative evidence (6, 36–38). There is an ever-expanding list of repair options, from autologous grafts to synthetic glues and even 3D-printed custom implants, without a complimentary expansion in the evidence base (6, 36, 39). These repair materials are sometimes supported by CSF diversion to reduce the pressure across the surgical repair. In fact, the only high-level evidence within the field of endonasal skull base repair is a randomized controlled trial investigating perioperative lumbar drainage (combined with nasoseptal flap repair) in EEA with high-flow intraoperative CSF leak (35). Lumbar drains were inserted immediately postoperatively (under the same anaesthetic), draining 10 ml/h for 3 days, resulting in a decrease in CSF rhinorrhoea rates (8.2% with lumbar drainage vs. 21.2% without; p = 0.03) (35).

Furthermore, most modern protocols adapt the extent of skull base reconstruction to postoperative CSF rhinorrhoea risk, balancing the risks of the former against the latter (4, 6, 10, 40–46). Numerous factors weigh into this decision-making, from demographics, co-morbidities, tumour characteristics, and operative factors (e.g., CSF leak), although the exact contribution of each potential factor in surgical decision-making remains poorly defined (6, 14, 22, 32, 44, 47, 48). Techniques reported commonly for low-risk cases include fat grafts, fascia lata grafts and synthetic grafts; whereas multilayer regimes with vascularized flaps, gasket-sealing, and lumbar drains are commoner in higher-risk cases (6, 37, 49, 50). Future studies would benefit from multimodal datasets which encompass these risk factors (e.g. combination of clinical metadata, imaging and operative video) and advanced analysis techniques (e.g. machine learning) to explore the interactions between risk factors, repair techniques and CSF rhinorrhoea rates.

### Strengths and limitations

The strengths of this study are its prospective, consecutive recruitment (despite COVID-19), and the creation of a collaborative network of neurosurgeons and otorhinolaryngologists with a specialist interest in skull-base and pituitary, spanning almost every adult neurosurgical centre in the UK and Ireland. There are several limitations. Firstly, the study involved only two countries, limiting the generalizability of the findings. Furthermore, the study is observational and occurred during a pandemic wave, possibly hampering case recruitment. Due to pandemic-related pressures and redeployments, several centres uploaded data in retrospect but submitted cases were reviewed in detail by supervising consultants. Only one dedicated pediatric centre was included, although 6 centres (joint adult and pediatric) included patients less than 16 years old. CSF rhinorrhoea was infrequent, whilst there was a wide array of combinations for relevant variables (particularly skull-base repair methods) making statistical analysis challenging.

## Conclusions

Heterogeneity of skull-base repair techniques exists across centres. Multilayer regimes with vascularized flaps, CSF diversion and rigid buttresses appear commoner in higher-risk cases, such as in EEAs. Overall, corresponding CSF rhinorrhoea rates across the UK and Ireland are lower than generally reported in the literature. A large proportion of postoperative leaks occurred in the context of occult intraoperative CSF leaks, and decisions for universal sellar repairs should consider the risks and cost-effectiveness of repair methods used. Future work could include longer-term, higher-volume studies, such as a registry; and high-quality interventional studies.

## Data availability statement

Data is available upon reasonable request. Requests to access the datasets should be directed to h.marcus@ucl.ac.uk.

## Ethics statement

Ethical review and approval was not required for the study on human participants in accordance with the local legislation and institutional requirements. Written informed consent for participation was not required for this study in accordance with the national legislation and the institutional requirements.

## CRANIAL Consortium

**Danyal Z Khan**, Department of Neurosurgery, National Hospital for Neurology and Neurosurgery, London; **Hani J Marcus**, Department of Neurosurgery, National Hospital for Neurology and Neurosurgery, London; **Soham Bandyopadhyay**, Oxford University Global Surgery Group, Nuffield Department of Surgical Sciences, University of Oxford, Oxford; **Benjamin E Schroeder**, Department of Neurology, University Hospital of Wales, Cardiff University, Cardiff; **Vikesh Patel**, Division of Neurosurgery, Cambridge University Hospitals Trust, Cambridge; **Alice O’Donnell**, Birmingham Medical School, University of Birmingham, Birmingham; **Neurology and Neurosurgery Interest Group**, NANSIG; **British Neurosurgical Trainee Research Collaborative**, BNTRC; **Anastasios Giamouriadis**, Department of Neurosurgery, Aberdeen Royal Infirmary, Aberdeen; **Pragnesh Bhatt**, Department of Neurosurgery, Aberdeen Royal Infirmary, Aberdeen; **Bhaskar Ram**, Department of Otorhinolaryngology, Aberdeen Royal Infirmary, Aberdeen; **Adithya Varma**, Department of Neurosurgery, Aberdeen Royal Infirmary, Aberdeen; **Philip Weir**, Department of Neurosurgery, Royal Victoria Hospital, Belfast; **Brendan Hanna**, Department of Otorhinolaryngology, Royal Victoria Hospital, Belfast; **Theodore C Hirst**, Department of Neurosurgery, Royal Victoria Hospital, Belfast; **Patrick McAleavey**, Department of Neurosurgery, Royal Victoria Hospital, Belfast; **Alessandro Paluzzi**, Department of Neurosurgery, Queen Elizabeth Hospital Birmingham, Birmingham; **Georgios Tsermoulas**, Department of Neurosurgery, Queen Elizabeth Hospital Birmingham, Birmingham; **Shahzada Ahmed**, Department of Otorhinolaryngology, Queen Elizabeth Hospital Birmingham, Birmingham; **Wai Cheong Soon**, Department of Neurosurgery, Queen Elizabeth Hospital Birmingham, Birmingham; **Yasir Arafat Chowdhury**, Department of Neurosurgery, Queen Elizabeth Hospital Birmingham, Birmingham; **Suhaib Abualsaud**, Department of Neurosurgery, Queen Elizabeth Hospital Birmingham, Birmingham; **Shumail Mahmood**, Department of Neurosurgery, Queen Elizabeth Hospital Birmingham, Birmingham; **Paresh Naik**, Department of Otorhinolaryngology, Queen Elizabeth Hospital Birmingham, Birmingham; **Zohra Haiderkhan**, Department of Neurosurgery, Queen Elizabeth Hospital Birmingham, Birmingham; **Rafid Al-Mahfoudh**, Department of Neurosurgery, Hurstwood Park Neurosciences Centre and Royal Sussex County Hospital, Brighton; **Andrea Perera**, Department of Neurosurgery, Hurstwood Park Neurosciences Centre and Royal Sussex County Hospital, Brighton; **Mircea Rus**, Department of Neurosurgery, Hurstwood Park Neurosciences Centre and Royal Sussex County Hospital, Brighton; **Adam Williams**, Department of Neurosurgery, Southmead Hospital Bristol, Bristol; **Charles Hand**, Department of Neurosurgery, Southmead Hospital Bristol, Bristol; **Kumar Abhinav**, Department of Neurosurgery, Southmead Hospital Bristol, Bristol; **Cristina Cernei**, Department of Neurosurgery, Southmead Hospital Bristol, Bristol; **Aiman Dilnawaz**, Department of Neurosurgery, Southmead Hospital Bristol, Bristol; **Richard Mannion**, Division of Neurosurgery, Cambridge University Hospitals Trust, Cambridge; **Thomas Santarius**, Division of Neurosurgery, Cambridge University Hospitals Trust, Cambridge; **James Tysome**, Division of Otorhinolaryngology, Cambridge University Hospitals Trust, Cambridge; **Rishi Sharma**, Division of Otorhinolaryngology, Cambridge University Hospitals Trust, Cambridge; **Angelos G Kolias**, Division of Neurosurgery, Cambridge University Hospitals Trust, Cambridge; **Neil Donnelly**, Division of Otorhinolaryngology, Cambridge University Hospitals Trust, Cambridge; **Vikesh Patel**, Division of Neurosurgery, Cambridge University Hospitals Trust, Cambridge; **Ashwin Venkatesh**, Division of Neurosurgery, Cambridge University Hospitals Trust, Cambridge; **Caroline Hayhurst**, Department of Neurosurgery, University Hospital of Wales, Cardiff; **Amr Mohamed**, Department of Neurosurgery, University Hospital of Wales, Cardiff; **Benjamin Stew**, Department of Otorhinolaryngology, University Hospital of Wales, Cardiff; **Joseph Merola**, Department of Neurosurgery, University Hospital of Wales, Cardiff; **Setthasorn Zhi Yang Ooi**, Department of Neurosurgery, University Hospital of Wales, Cardiff; **Mahmoud Kamel**, Department of Neurosurgery, Cork University Hospitals, Ireland; **Mohammad Habibullah Khan**, Department of Otorhinolaryngology, Cork University Hospitals, Ireland; **Sahibzada Abrar**, Department of Neurosurgery, Cork University Hospitals, Ireland; **Christopher Mckeon**, Department of Neurosurgery, Cork University Hospitals, Ireland; **Daniel McSweeney**, Department of Neurosurgery, Cork University Hospitals, Ireland; **Mohsen Javadpour**, Department of Neurosurgery, National Neurosurgical Centre, Beaumont Hospital, Ireland; **Peter Lacy**, Department of Otorhinolaryngology, National Neurosurgical Centre, Beaumont Hospital, Ireland; **Daniel Murray**, Department of Neurosurgery, National Neurosurgical Centre, Beaumont Hospital, Ireland; **Elena Roman**, Department of Neurosurgery, National Neurosurgical Centre, Beaumont Hospital, Ireland; **Kismet Hossain-Ibrahim**, Department of Neurosurgery, Ninewells Hospital, Dundee; **Peter Ross**, Department of Otorhinolaryngology, Ninewells Hospital, Dundee; **David Bennett**, Department of Neurosurgery, Ninewells Hospital, Dundee; **Nathan McSorley**, Department of Neurosurgery, Ninewells Hospital, Dundee; **Adam Hounat**, Department of Neurosurgery, Ninewells Hospital, Dundee; **Patrick Statham**, Department of Clinical Neurosciences, BioQuarter, Edinburgh; **Mark Hughes**, Department of Clinical Neurosciences, BioQuarter, Edinburgh; **Alhafidz Hamdan**, Department of Clinical Neurosciences, BioQuarter, Edinburgh; **Caroline Scott**, Department of Clinical Neurosciences, BioQuarter, Edinburgh; **Jigi Moudgil-Joshi**, Department of Clinical Neurosciences, BioQuarter, Edinburgh; **Anuj Bahl**, Department of Neurosurgery, Hull University Teaching Hospitals, Hull; **Anna Bjornson**, Department of Neurosurgery, Hull University Teaching Hospitals, Hull; **Daniel Gatt**, Department of Neurosurgery, Hull University Teaching Hospitals, Hull; **Nick Phillips**, Department of Neurosurgery, Leeds Teaching Hospitals, Leeds; **Neeraj Kalra**, Department of Neurosurgery, Leeds Teaching Hospitals, Leeds; **Melissa Bautista**, Department of Neurosurgery, Leeds Teaching Hospitals, Leeds; **Seerat Shirazi**, Department of Neurosurgery, Leeds Teaching Hospitals, Leeds; **Catherine E Gilkes**, Department of Neurosurgery, The Walton Centre, Liverpool; **Christopher P Millward**, Department of Neurosurgery, The Walton Centre, Liverpool; **Ahmad MS Ali**, Department of Neurosurgery, The Walton Centre, Liverpool; **Dimitris Paraskevopoulos**, Department of Neurosurgery, Barts and The Royal London Hospital, London; **Jarnail Bal**, Department of Neurosurgery, Barts and The Royal London Hospital, London; **Samir Matloob**, Department of Neurosurgery, Barts and The Royal London Hospital, London; **Rhannon Lobo**, Department of Neurosurgery, Barts and The Royal London Hospital, London; **Nigel Mendoza**, Department of Neurosurgery, Charing Cross Hospital, London; **Ramesh Nair**, Department of Neurosurgery, Charing Cross Hospital, London; **Arthur Dalton**, Department of Neurosurgery, Charing Cross Hospital, London; **Adarsh Nadig**, Department of Neurosurgery, Charing Cross Hospital, London; **Lucas Hernandez**, Department of Neurosurgery, Charing Cross Hospital, London; **Nick Thomas**, Department of Neurosurgery, King's College Hospital, London; **Eleni Maratos**, Department of Neurosurgery, King's College Hospital, London; **Jonathan Shapey**, Department of Neurosurgery, King's College Hospital, London; **Sinan Al-Barazi**, Department of Neurosurgery, King's College Hospital, London; **Asfand Baig Mirza**, Department of Neurosurgery, King's College Hospital, London; **Mohamed Okasha**, Department of Neurosurgery, King's College Hospital, London; **Prabhjot Singh Malhotra**, Department of Neurosurgery, King's College Hospital, London; **Razna Ahmed**, Department of Neurosurgery, King's College Hospital, London; **Neil L Dorward**, Department of Neurosurgery, National Hospital for Neurology and Neurosurgery, London; **Joan Grieve**, Department of Neurosurgery, National Hospital for Neurology and Neurosurgery, London; **Parag Sayal**, Department of Neurosurgery, National Hospital for Neurology and Neurosurgery, London; **David Choi**, Department of Neurosurgery, National Hospital for Neurology and Neurosurgery, London; **Ivan Cabrilo**, Department of Neurosurgery, National Hospital for Neurology and Neurosurgery, London; **Hugo Layard Horsfall**, Department of Neurosurgery, National Hospital for Neurology and Neurosurgery, London; **Jonathan Pollock**, Department of Neurosurgery, Barking, Havering & Redbridge University Hospitals, London; **Alireza Shoakazemi**, Department of Neurosurgery, Barking, Havering & Redbridge University Hospitals, London; **Oscar Maccormac**, Department of Neurosurgery, Barking, Havering & Redbridge University Hospitals, London; **Guru N K Amirthalingam**, Department of Neurosurgery, Barking, Havering & Redbridge University Hospitals, London; **Andrew Martin**, Department of Neurosurgery, St George’s University Hospitals Trust, London; **Simon Stapleton**, Department of Neurosurgery, St George’s University Hospitals Trust, London; **Florence Hogg**, Department of Neurosurgery, St George’s University Hospitals Trust, London; **Daniel Richardson**, Department of Neurosurgery, St George’s University Hospitals Trust, London; **Kanna Gnanalingham**, Department of Neurosurgery, Salford Royal Trust, Manchester; **Omar Pathmanaban**, Department of Neurosurgery, Salford Royal Trust, Manchester; **Daniel M Fountain**, Department of Neurosurgery, Salford Royal Trust, Manchester; **Raj Bhalla**, Department of Otorhinolaryngology, Salford Royal Trust, Manchester; **Cathal J Hannan**, Department of Neurosurgery, Salford Royal Trust, Manchester; **Annabel Chadwick**, Department of Neurosurgery, Salford Royal Trust, Manchester; **Alistair Jenkins**, Department of Neurosurgery, Royal Victoria Infirmary, Newcastle; **Claire Nicholson**, Department of Neurosurgery, Royal Victoria Infirmary, Newcastle; **Syed Shumon**, Department of Neurosurgery, Royal Victoria Infirmary, Newcastle; **Mohamed Youssef**, Department of Neurosurgery, Royal Victoria Infirmary, Newcastle; **Callum Allison**, Department of Neurosurgery, Royal Victoria Infirmary, Newcastle; **Graham Dow**, Department of Neurosurgery, Queen's Medical Centre Nottingham, Nottingham; **Iain Robertson**, Department of Neurosurgery, Queen's Medical Centre Nottingham, Nottingham; **Laurence Johann Glancz**, Department of Neurosurgery, Queen's Medical Centre Nottingham, Nottingham; **Murugan Sitaraman**, Department of Neurosurgery, Queen's Medical Centre Nottingham, Nottingham; **Ashwin Kumaria**, Department of Neurosurgery, Queen's Medical Centre Nottingham, Nottingham; **Ananyo Bagchi**, Department of Neurosurgery, Queen's Medical Centre Nottingham, Nottingham; **Simon Cudlip**, Department of Neurosurgery, John Radcliffe Hospital, Oxford University Hospitals, Oxford; **Jane Halliday**, Department of Neurosurgery, John Radcliffe Hospital, Oxford University Hospitals, Oxford; **Rory J Piper**, Department of Neurosurgery, John Radcliffe Hospital, Oxford University Hospitals, Oxford; **Alexandros Boukas**, Department of Neurosurgery, John Radcliffe Hospital, Oxford University Hospitals, Oxford; **Meriem Amarouche**, Department of Neurosurgery, John Radcliffe Hospital, Oxford University Hospitals, Oxford; **Damjan Veljanoski**, Department of Neurosurgery, John Radcliffe Hospital, Oxford University Hospitals, Oxford; **Samiul Muquit**, Department of Neurosurgery, University Hospitals Plymouth, Plymouth; **Ellie Edlmann**, Department of Neurosurgery, University Hospitals Plymouth, Plymouth; **Haritha Maripi**, Department of Neurosurgery, University Hospitals Plymouth, Plymouth; **Yi Wang**, Department of Neurosurgery, University Hospitals Plymouth, Plymouth; **Mehnaz Hossain**, Department of Neurosurgery, University Hospitals Plymouth, Plymouth; **Andrew Alalade**, Department of Neurosurgery, Lancashire Teaching Hospitals NHS Foundation Trust, Preston; **Syed Maroof**, Department of Neurosurgery, Lancashire Teaching Hospitals NHS Foundation Trust, Preston; **Pradnya Patkar**, Department of Neurosurgery, Lancashire Teaching Hospitals NHS Foundation Trust, Preston; **Saurabh Sinha**, Department of Neurosurgery, Royal Hallamshire Hospital & Sheffield Children’s Hospital, Sheffield; **Showkat Mirza**, Department of Otorhinolaryngology, Royal Hallamshire Hospital & Sheffield Children’s Hospital, Sheffield; **Duncan Henderson**, Department of Neurosurgery, Royal Hallamshire Hospital & Sheffield Children’s Hospital, Sheffield; **Mohammad Saud Khan**, Department of Neurosurgery, Royal Hallamshire Hospital & Sheffield Children’s Hospital, Sheffield; **Nijaguna Mathad**, Department of Neurosurgery, University Hospital Southampton, Southampton; **Jonathan Hempenstall**, Department of Neurosurgery, University Hospital Southampton, Southampton; **Difei Wang,** Department of Neurosurgery, University Hospital Southampton, Southampton; **Pavan Marwaha**, Department of Neurosurgery, University Hospital Southampton, Southampton; **Simon Shaw**, Department of Neurosurgery, Royal Stoke University Hospital, Stoke; **Georgios Solomou**, Department of Neurosurgery, Royal Stoke University Hospital, Stoke; **Alina Shrestha**, Department of Neurosurgery, Royal Stoke University Hospital, Stoke. List of Collaborators (data validators): **Andrew Fraser**, Department of Neurosurgery, Aberdeen Royal Infirmary, Aberdeen; **Theodore Hirst**, Department of Neurosurgery, Royal Victoria Hospital, Belfast; **Yasir Chowdhury,** Department of Neurosurgery, Queen Elizabeth Hospital Birmingham, Birmingham; **Sobiya Bilal**, Department of Neurosurgery, Hurstwood Park Neurosciences Centre and Royal Sussex County Hospital, Brighton; **Jack Wildman**, Department of Neurosurgery, Southmead Hospital Bristol, Bristol; **Ashwin Venkatesh**, Division of Neurosurgery, Cambridge University Hospitals Trust, Cambridge; **Priya Babu**, Department of Neurosurgery, University Hospital of Wales, Cardiff; **Cian Carey**, Department of Neurosurgery, Cork University Hospitals, Ireland; **Renitha Reddi Bathuni**, Department of Neurosurgery, National Neurosurgical Centre, Beaumont Hospital, Ireland; **Kismet Hossain-Ibrahim**, Department of Neurosurgery, Ninewells Hospital, Dundee; **Joseph Nathaniel Brennan**, Department of Neurosurgery, The Western General Hospital, Edinburgh; **Anna Bjornson**, Department of Neurosurgery, Hull University Teaching Hospitals, Hull **Howra Ktayen**, Department of Neurosurgery, Leeds Teaching Hospitals, Leeds; **Sandhya T Trichinopoly**, Department of Neurosurgery, The Walton Centre, Liverpool; **Samir Matloob**, Department of Neurosurgery, Barts and The Royal London Hospital, London; **Adarsh Nadig**, Department of Neurosurgery, Charing Cross Hospital, London; **Mohamed Okasha,** Department of Neurosurgery, King's College Hospital, London; **Danyal Khan**, Department of Neurosurgery, National Hospital for Neurology and Neurosurgery, London; **Alireza Shoakazemi**, Department of Neurosurgery, Barking, Havering & Redbridge University Hospitals, London; **Florence Hogg**, Department of Neurosurgery, St George’s University Hospitals Trust, London; **Seun Sobawale**, Department of Neurosurgery, Salford Royal Trust, Manchester; **Amir Suliman,** Department of Neurosurgery, Royal Victoria Infirmary, Newcastle; **Ashwin Kumaria**, Department of Neurosurgery, Queen's Medical Centre Nottingham, Nottingham; **Rory Piper**, Department of Neurosurgery, John Radcliffe Hospital, Oxford University Hospitals, Oxford; **Will Owen**, Department of Neurosurgery, John Radcliffe Hospital, Oxford University Hospitals, Oxford; **Ellie Edlmann**, Department of Neurosurgery, University Hospitals Plymouth, Plymouth; **Afaq Sartaj**, Department of Neurosurgery, Lancashire Teaching Hospitals NHS Foundation Trust, Preston; **Edward Goacher**, Department of Neurosurgery, Royal Hallamshire Hospital & Sheffield Children’s Hospital, Sheffield; **Euan Strachan**, Department of Neurosurgery, University Hospital Southampton, Southampton; **Giorgios Solomou**, Department of Neurosurgery, Royal Stoke University Hospital, Stoke.

## Author contributions

This is a group authorship model paper where all authors contributed to data collection and approved the final manuscript. All authors listed have made a substantial, direct, and intellectual contribution to the work and approved it for publication.

## Acknowledgments

The authors would like to thank the Neurology and Neurosurgery Interest Group (NANSIG) and the British Neurosurgical Trainee Research Collaborative (BNTRC) without which this study would not have been possible. A special thanks to the data validation team for ensuring data accuracy (**Supplementary Material 1**).

## Conflict of interest

HM is supported by the Wellcome (203145Z/16/Z) EPSRC (NS/A000050/1) Centre for Interventional and Surgical Sciences, University College London. HM is also funded by the NIHR Biomedical Research Centre at University College London. DK is supported by an NIHR Academic Clinical Fellowship. DK is also supported by a Cancer Research UK Predoctoral Fellowship. For the purpose of Open Access, the authors have applied a CC BY public copyright license to any Author Accepted Manuscript version arising from this submission.

The remaining authors declare that the research was conducted in the absence of any commercial or financial relationships that could be construed as a potential conflict of interest.

## Publisher’s note

All claims expressed in this article are solely those of the authors and do not necessarily represent those of their affiliated organizations, or those of the publisher, the editors and the reviewers. Any product that may be evaluated in this article, or claim that may be made by its manufacturer, is not guaranteed or endorsed by the publisher.
