# Supplementary material for: CSF rhinorrhoea after endonasal intervention to the skull base (CRANIAL): A multicentre prospective observational study
Source: Front Oncol. 2023 Jan 4;12:1049627. doi: 10.3389/fonc.2022.1049627 (PMC9846732; doi:10.3389/fonc.2022.1049627)
Supplement: Supplementary file 1 [file DataSheet_1.docx]

**Supplementary Materials:**

Supplementary material 1: List of authors and collaborators

1a. Authors

| **Team** | **Name** |
| --- | --- |
| Department of Neurosurgery, National Hospital for Neurology and Neurosurgery, London | Danyal Z Khan |
| Department of Neurosurgery, National Hospital for Neurology and Neurosurgery, London | Hani J Marcus |
| Oxford University Global Surgery Group, Nuffield Department of Surgical Sciences, University of Oxford, Oxford | Soham Bandyopadhyay |
| Department of Neurology, University Hospital of Wales, Cardiff University, Cardiff | Benjamin E Schroeder |
| Division of Neurosurgery, Cambridge University Hospitals Trust, Cambridge | Vikesh Patel |
| Birmingham Medical School, University of Birmingham, Birmingham | Alice O’Donnell |
| NANSIG | Neurology and Neurosurgery Interest Group |
| BNTRC | British Neurosurgical Trainee Research Collaborative |
| Department of Neurosurgery, Aberdeen Royal Infirmary, Aberdeen | Anastasios Giamouriadis |
| Department of Neurosurgery, Aberdeen Royal Infirmary, Aberdeen | Pragnesh Bhatt |
| Department of Otorhinolaryngology, Aberdeen Royal Infirmary, Aberdeen | Bhaskar Ram |
| Department of Neurosurgery, Aberdeen Royal Infirmary, Aberdeen | Adithya Varma |
| Department of Neurosurgery, Royal Victoria Hospital, Belfast | Philip Weir |
| Department of Otorhinolaryngology, Royal Victoria Hospital, Belfast | Brendan Hanna |
| Department of Neurosurgery, Royal Victoria Hospital, Belfast | Theodore C Hirst |
| Department of Neurosurgery, Royal Victoria Hospital, Belfast | Patrick McAleavey |
| Department of Neurosurgery, Queen Elizabeth Hospital Birmingham, Birmingham | Alessandro Paluzzi |
| Department of Neurosurgery, Queen Elizabeth Hospital Birmingham, Birmingham | Georgios Tsermoulas |
| Department of Otorhinolaryngology, Queen Elizabeth Hospital Birmingham, Birmingham | Shahzada Ahmed |
| Department of Neurosurgery, Queen Elizabeth Hospital Birmingham, Birmingham | Wai Cheong Soon |
| Department of Neurosurgery, Queen Elizabeth Hospital Birmingham, Birmingham | Yasir Arafat Chowdhury |
| Department of Neurosurgery, Queen Elizabeth Hospital Birmingham, Birmingham | Suhaib Abualsaud |
| Department of Neurosurgery, Queen Elizabeth Hospital Birmingham, Birmingham | Shumail Mahmood |
| Department of Otorhinolaryngology, Queen Elizabeth Hospital Birmingham, Birmingham | Paresh Naik |
| Department of Neurosurgery, Queen Elizabeth Hospital Birmingham, Birmingham | Zohra Haiderkhan |
| Department of Neurosurgery, Hurstwood Park Neurosciences Centre and Royal Sussex County Hospital, Brighton | Rafid Al-Mahfoudh |
| Department of Neurosurgery, Hurstwood Park Neurosciences Centre and Royal Sussex County Hospital, Brighton | Andrea Perera |
| Department of Neurosurgery, Hurstwood Park Neurosciences Centre and Royal Sussex County Hospital, Brighton | Mircea Rus |
| Department of Neurosurgery, Southmead Hospital Bristol, Bristol | Adam Williams |
| Department of Neurosurgery, Southmead Hospital Bristol, Bristol | Charles Hand |
| Department of Neurosurgery, Southmead Hospital Bristol, Bristol | Kumar Abhinav |
| Department of Neurosurgery, Southmead Hospital Bristol, Bristol | Cristina Cernei |
| Department of Neurosurgery, Southmead Hospital Bristol, Bristol | Aiman Dilnawaz |
| Division of Neurosurgery, Cambridge University Hospitals Trust, Cambridge | Richard Mannion |
| Division of Neurosurgery, Cambridge University Hospitals Trust, Cambridge | Thomas Santarius |
| Division of Otorhinolaryngology, Cambridge University Hospitals Trust, Cambridge | James Tysome |
| Division of Otorhinolaryngology, Cambridge University Hospitals Trust, Cambridge | Rishi Sharma |
| Division of Neurosurgery, Cambridge University Hospitals Trust, Cambridge | Angelos G Kolias |
| Division of Otorhinolaryngology, Cambridge University Hospitals Trust, Cambridge | Neil Donnelly |
| Division of Neurosurgery, Cambridge University Hospitals Trust, Cambridge | Vikesh Patel |
| Division of Neurosurgery, Cambridge University Hospitals Trust, Cambridge | Ashwin Venkatesh |
| Department of Neurosurgery, University Hospital of Wales, Cardiff | Caroline Hayhurst |
| Department of Neurosurgery, University Hospital of Wales, Cardiff | Amr Mohamed |
| Department of Otorhinolaryngology, University Hospital of Wales, Cardiff | Benjamin Stew |
| Department of Neurosurgery, University Hospital of Wales, Cardiff | Joseph Merola |
| Department of Neurosurgery, University Hospital of Wales, Cardiff | Setthasorn Zhi Yang Ooi |
| Department of Neurosurgery, Cork University Hospitals, Ireland | Mahmoud Kamel |
| Department of Otorhinolaryngology, Cork University Hospitals, Ireland | Mohammad Habibullah Khan |
| Department of Neurosurgery, Cork University Hospitals, Ireland | Sahibzada Abrar |
| Department of Neurosurgery, Cork University Hospitals, Ireland | Christopher Mckeon |
| Department of Neurosurgery, Cork University Hospitals, Ireland | Daniel McSweeney |
| Department of Neurosurgery, National Neurosurgical Centre, Beaumont Hospital, Ireland | Mohsen Javadpour |
| Department of Otorhinolaryngology, National Neurosurgical Centre, Beaumont Hospital, Ireland | Peter Lacy |
| Department of Neurosurgery, National Neurosurgical Centre, Beaumont Hospital, Ireland | Daniel Murray |
| Department of Neurosurgery, National Neurosurgical Centre, Beaumont Hospital, Ireland | Elena Roman |
| Department of Neurosurgery, Ninewells Hospital, Dundee | Kismet Hossain-Ibrahim |
| Department of Otorhinolaryngology, Ninewells Hospital, Dundee | Peter Ross |
| Department of Neurosurgery, Ninewells Hospital, Dundee | David Bennett |
| Department of Neurosurgery, Ninewells Hospital, Dundee | Nathan McSorley |
| Department of Neurosurgery, Ninewells Hospital, Dundee | Adam Hounat |
| Department of Clinical Neurosciences, BioQuarter, Edinburgh | Patrick Statham |
| Department of Clinical Neurosciences, BioQuarter, Edinburgh | Mark Hughes |
| Department of Clinical Neurosciences, BioQuarter, Edinburgh | Alhafidz Hamdan |
| Department of Clinical Neurosciences, BioQuarter, Edinburgh | Caroline Scott |
| Department of Clinical Neurosciences, BioQuarter, Edinburgh | Jigi Moudgil-Joshi |
| Department of Neurosurgery, Hull University Teaching Hospitals, Hull | Anuj Bahl |
| Department of Neurosurgery, Hull University Teaching Hospitals, Hull | Anna Bjornson |
| Department of Neurosurgery, Hull University Teaching Hospitals, Hull | Daniel Gatt |
| Department of Neurosurgery, Leeds Teaching Hospitals, Leeds | Nick Phillips |
| Department of Neurosurgery, Leeds Teaching Hospitals, Leeds | Neeraj Kalra |
| Department of Neurosurgery, Leeds Teaching Hospitals, Leeds | Melissa Bautista |
| Department of Neurosurgery, Leeds Teaching Hospitals, Leeds | Seerat Shirazi |
| Department of Neurosurgery, The Walton Centre, Liverpool | Catherine E Gilkes |
| Department of Neurosurgery, The Walton Centre, Liverpool | Christopher P Millward |
| Department of Neurosurgery, The Walton Centre, Liverpool | Ahmad MS Ali |
| Department of Neurosurgery, Barts and The Royal London Hospital, London | Dimitris Paraskevopoulos |
| Department of Neurosurgery, Barts and The Royal London Hospital, London | Jarnail Bal |
| Department of Neurosurgery, Barts and The Royal London Hospital, London | Samir Matloob |
| Department of Neurosurgery, Barts and The Royal London Hospital, London | Rhannon Lobo |
| Department of Neurosurgery, Charing Cross Hospital, London | Nigel Mendoza |
| Department of Neurosurgery, Charing Cross Hospital, London | Ramesh Nair |
| Department of Neurosurgery, Charing Cross Hospital, London | Arthur Dalton |
| Department of Neurosurgery, Charing Cross Hospital, London | Adarsh Nadig |
| Department of Neurosurgery, Charing Cross Hospital, London | Lucas Hernandez |
| Department of Neurosurgery, King's College Hospital, London | Nick Thomas |
| Department of Neurosurgery, King's College Hospital, London | Eleni Maratos |
| Department of Neurosurgery, King's College Hospital, London | Jonathan Shapey |
| Department of Neurosurgery, King's College Hospital, London | Sinan Al-Barazi |
| Department of Neurosurgery, King's College Hospital, London | Asfand Baig Mirza |
| Department of Neurosurgery, King's College Hospital, London | Mohamed Okasha |
| Department of Neurosurgery, King's College Hospital, London | Prabhjot Singh Malhotra |
| Department of Neurosurgery, King's College Hospital, London | Razna Ahmed |
| Department of Neurosurgery, National Hospital for Neurology and Neurosurgery, London | Neil L Dorward |
| Department of Neurosurgery, National Hospital for Neurology and Neurosurgery, London | Joan Grieve |
| Department of Neurosurgery, National Hospital for Neurology and Neurosurgery, London | Parag Sayal |
| Department of Neurosurgery, National Hospital for Neurology and Neurosurgery, London | David Choi |
| Department of Neurosurgery, National Hospital for Neurology and Neurosurgery, London | Ivan Cabrilo |
| Department of Neurosurgery, National Hospital for Neurology and Neurosurgery, London | Hugo Layard Horsfall |
| Department of Neurosurgery, Barking, Havering & Redbridge University Hospitals, London | Jonathan Pollock |
| Department of Neurosurgery, Barking, Havering & Redbridge University Hospitals, London | Alireza Shoakazemi |
| Department of Neurosurgery, Barking, Havering & Redbridge University Hospitals, London | Oscar Maccormac |
| Department of Neurosurgery, Barking, Havering & Redbridge University Hospitals, London | Guru N K Amirthalingam |
| Department of Neurosurgery, St George’s University Hospitals Trust, London | Andrew Martin |
| Department of Neurosurgery, St George’s University Hospitals Trust, London | Simon Stapleton |
| Department of Neurosurgery, St George’s University Hospitals Trust, London | Florence Hogg |
| Department of Neurosurgery, St George’s University Hospitals Trust, London | Daniel Richardson |
| Department of Neurosurgery, Salford Royal Trust, Manchester | Kanna Gnanalingham |
| Department of Neurosurgery, Salford Royal Trust, Manchester | Omar Pathmanaban |
| Department of Neurosurgery, Salford Royal Trust, Manchester | Daniel M Fountain |
| Department of Otorhinolaryngology, Salford Royal Trust, Manchester | Raj Bhalla |
| Department of Neurosurgery, Salford Royal Trust, Manchester | Cathal J Hannan |
| Department of Neurosurgery, Salford Royal Trust, Manchester | Annabel Chadwick |
| Department of Neurosurgery, Royal Victoria Infirmary, Newcastle | Alistair Jenkins |
| Department of Neurosurgery, Royal Victoria Infirmary, Newcastle | Claire Nicholson |
| Department of Neurosurgery, Royal Victoria Infirmary, Newcastle | Syed Shumon |
| Department of Neurosurgery, Royal Victoria Infirmary, Newcastle | Mohamed Youssef |
| Department of Neurosurgery, Royal Victoria Infirmary, Newcastle | Callum Allison |
| Department of Neurosurgery, Queen's Medical Centre Nottingham, Nottingham | Graham Dow |
| Department of Neurosurgery, Queen's Medical Centre Nottingham, Nottingham | Iain Robertson |
| Department of Neurosurgery, Queen's Medical Centre Nottingham, Nottingham | Laurence Johann Glancz |
| Department of Neurosurgery, Queen's Medical Centre Nottingham, Nottingham | Murugan Sitaraman |
| Department of Neurosurgery, Queen's Medical Centre Nottingham, Nottingham | Ashwin Kumaria |
| Department of Neurosurgery, Queen's Medical Centre Nottingham, Nottingham | Ananyo Bagchi |
| Department of Neurosurgery, John Radcliffe Hospital, Oxford University Hospitals, Oxford | Simon Cudlip |
| Department of Neurosurgery, John Radcliffe Hospital, Oxford University Hospitals, Oxford | Jane Halliday |
| Department of Neurosurgery, John Radcliffe Hospital, Oxford University Hospitals, Oxford | Rory J Piper |
| Department of Neurosurgery, John Radcliffe Hospital, Oxford University Hospitals, Oxford | Alexandros Boukas |
| Department of Neurosurgery, John Radcliffe Hospital, Oxford University Hospitals, Oxford | Meriem Amarouche |
| Department of Neurosurgery, John Radcliffe Hospital, Oxford University Hospitals, Oxford | Damjan Veljanoski |
| Department of Neurosurgery, University Hospitals Plymouth, Plymouth | Samiul Muquit |
| Department of Neurosurgery, University Hospitals Plymouth, Plymouth | Ellie Edlmann |
| Department of Neurosurgery, University Hospitals Plymouth, Plymouth | Haritha Maripi |
| Department of Neurosurgery, University Hospitals Plymouth, Plymouth | Yi Wang |
| Department of Neurosurgery, University Hospitals Plymouth, Plymouth | Mehnaz Hossain |
| Department of Neurosurgery, Lancashire Teaching Hospitals NHS Foundation Trust, Preston | Andrew Alalade |
| Department of Neurosurgery, Lancashire Teaching Hospitals NHS Foundation Trust, Preston | Syed Maroof |
| Department of Neurosurgery, Lancashire Teaching Hospitals NHS Foundation Trust, Preston | Pradnya Patkar |
| Department of Neurosurgery, Royal Hallamshire Hospital & Sheffield Children’s Hospital, Sheffield | Saurabh Sinha |
| Department of Otorhinolaryngology, Royal Hallamshire Hospital & Sheffield Children’s Hospital, Sheffield | Showkat Mirza |
| Department of Neurosurgery, Royal Hallamshire Hospital & Sheffield Children’s Hospital, Sheffield | Duncan Henderson |
| Department of Neurosurgery, Royal Hallamshire Hospital & Sheffield Children’s Hospital, Sheffield | Mohammad Saud Khan |
| Department of Neurosurgery, University Hospital Southampton, Southampton | Nijaguna Mathad |
| Department of Neurosurgery, University Hospital Southampton, Southampton | Jonathan Hempenstall |
| Department of Neurosurgery, University Hospital Southampton, Southampton | Difei Wang |
| Department of Neurosurgery, University Hospital Southampton, Southampton | Pavan Marwaha |
| Department of Neurosurgery, Royal Stoke University Hospital, Stoke | Simon Shaw |
| Department of Neurosurgery, Royal Stoke University Hospital, Stoke | Georgios Solomou |
| Department of Neurosurgery, Royal Stoke University Hospital, Stoke | Alina Shrestha |

1b. Collaborators (data validators)

| **Team** | **Name** |
| --- | --- |
| Department of Neurosurgery, Aberdeen Royal Infirmary, Aberdeen | Andrew Fraser |
| Department of Neurosurgery, Royal Victoria Hospital, Belfast | Theodore Hirst |
| Department of Neurosurgery, Queen Elizabeth Hospital Birmingham, Birmingham | Yasir Chowdhury |
| Department of Neurosurgery, Hurstwood Park Neurosciences Centre and Royal Sussex County Hospital, Brighton | Sobiya Bilal |
| Department of Neurosurgery, Southmead Hospital Bristol, Bristol | Jack Wildman |
| Division of Neurosurgery, Cambridge University Hospitals Trust, Cambridge | Ashwin Venkatesh |
| Department of Neurosurgery, University Hospital of Wales, Cardiff | Priya Babu |
| Department of Neurosurgery, Cork University Hospitals, Ireland | Cian Carey |
| Department of Neurosurgery, National Neurosurgical Centre, Beaumont Hospital, Ireland | Renitha Reddi Bathuni |
| Department of Neurosurgery, Ninewells Hospital, Dundee | Kismet Hossain-Ibrahim |
| Department of Neurosurgery, The Western General Hospital, Edinburgh | Joseph Nathaniel Brennan |
| Department of Neurosurgery, Hull University Teaching Hospitals, Hull | Anna Bjornson |
| Department of Neurosurgery, Leeds Teaching Hospitals, Leeds | Howra Ktayen |
| Department of Neurosurgery, The Walton Centre, Liverpool | Sandhya T Trichinopoly |
| Department of Neurosurgery, Barts and The Royal London Hospital, London | Samir Matloob |
| Department of Neurosurgery, Charing Cross Hospital, London | Adarsh Nadig |
| Department of Neurosurgery, King's College Hospital, London | Mohamed Okasha |
| Department of Neurosurgery, National Hospital for Neurology and Neurosurgery, London | Danyal Khan |
| Department of Neurosurgery, Barking, Havering & Redbridge University Hospitals, London | Alireza Shoakazemi |
| Department of Neurosurgery, St George’s University Hospitals Trust, London | Florence Hogg |
| Department of Neurosurgery, Salford Royal Trust, Manchester | Seun Sobawale |
| Department of Neurosurgery, Royal Victoria Infirmary, Newcastle | Amir Suliman |
| Department of Neurosurgery, Queen's Medical Centre Nottingham, Nottingham | Ashwin Kumaria |
| Department of Neurosurgery, John Radcliffe Hospital, Oxford University Hospitals, Oxford | Rory Piper |
| Department of Neurosurgery, John Radcliffe Hospital, Oxford University Hospitals, Oxford | Will Owen |
| Department of Neurosurgery, University Hospitals Plymouth, Plymouth | Ellie Edlmann |
| Department of Neurosurgery, Lancashire Teaching Hospitals NHS Foundation Trust, Preston | Afaq Sartaj |
| Department of Neurosurgery, Royal Hallamshire Hospital & Sheffield Children’s Hospital, Sheffield | Edward Goacher |
| Department of Neurosurgery, University Hospital Southampton, Southampton | Euan Strachan |
| Department of Neurosurgery, Royal Stoke University Hospital, Stoke | Giorgios Solomou |

Supplementary material 2: Levels for skull base repair from which study repair technique taxonomy was derived. Adapted with permission from: Skull base repair following endonasal pituitary and skull base tumour resection: a systematic review, Pituitary, 2021, Khan DZ et al.


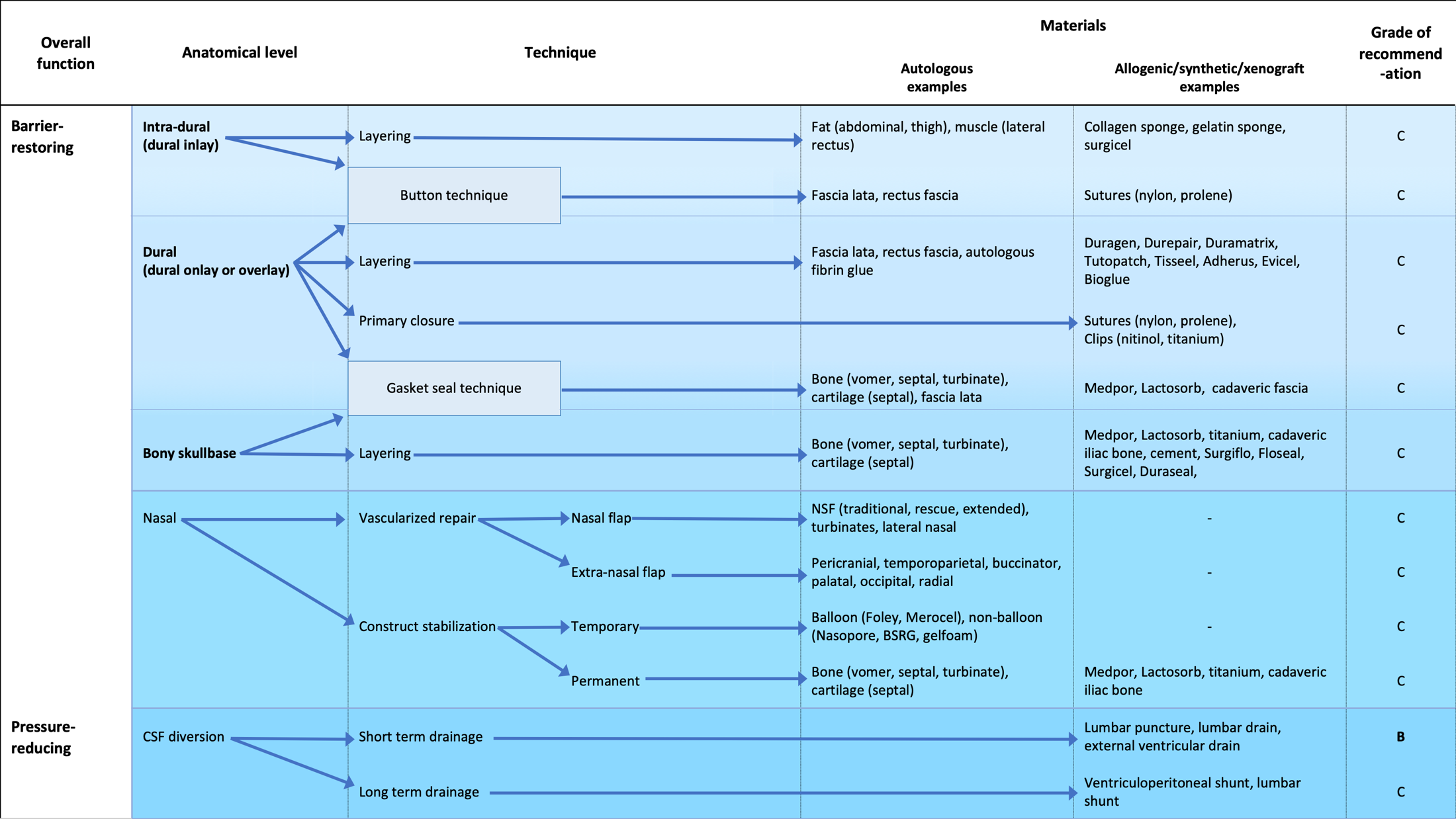


Supplementary material 3: Table of tumour types included by approach.

| **Row Labels** | **Transsphenoidal Approach** | **Expanded Endoscopic Endonasal Approach** | **Grand Total** |
| --- | --- | --- | --- |
| Apoplexy | 7 (1.0%) | 1 (0.7%) | 8 (0.9%) |
| Arachnoid cyst | 3 (0.4%) | 1 (0.7%) | 4 (0.5%) |
| Chordoma | 0 (0%) | 15 (10.7%) | 15 (1.7%) |
| Craniopharyngioma | 3 (0.4%) | 38 (27.1%) | 41 (4.7%) |
| Dermoid cyst | 0 (0%) | 1 (0.7%) | 1 (0.1%) |
| Germinoma | 1 (0.1%) | 0 (0%) | 1 (0.1%) |
| Hypophysitis | 1 (0.1%) | 0 (0%) | 1 (0.1%) |
| Meningioma | 3 (0.4%) | 25 (17.9%) | 28 (3.2%) |
| Meningoencephalocele | 0 (0%) | 1 (0.7%) | 1 (0.1%) |
| Neuroendocrine tumour | 1 (0.1%) | 0 (0%) | 1 (0.1%) |
| Other | 3 (0.4%) | 1 (0.7%) | 4 (0.5%) |
| Pituitary adenoma (Cushing’s) | 249 (34.3%) | 14 (10.0%) | 69 (8%) |
| Pituitary adenoma (Non-functioning) | 410 (56.5%) | 23 (16.4%) | 433 (50%) |
| Rathke's Cleft Cyst | 26 (3.6%) | 2 (1.4%) | 28 (3.2%) |
| Sinonasal endocrine tumour | 0 (0%) | 1 (0.7%) | 1 (0.1%) |
| Squamous cell carcinoma | 0 (0%) | 1 (0.7%) | 1 (0.1%) |
| Lymphocytic Hypophysitis | 6 (0.8%) | 0 (0%) | 6 (0.7%) |
| Mucocele | 1 (0.1%) | 0 (0%) | 1 (0.1%) |
| Epidermoid cyst | 1 (0.1%) | 0 (0%) | 1 (0.1%) |
| Pituitary abscess | 2 (0.3%) | 0 (0%) | 2 (0.2%) |
| Low grade spindle cell sarcomatous tumour | 1 (0.1%) | 0 (0%) | 1 (0.1%) |
| Simple cyst | 1 (0.1%) | 0 (0%) | 1 (0.1%) |
| Sellar Rhabdoid | 1 (0.1%) | 0 (0%) | 1 (0.1%) |
| Cyst (Uncertain aetiology) | 1 (0.1%) | 0 (0%) | 1 (0.1%) |
| Pituicytoma | 1 (0.1%) | 0 (0%) | 1 (0.1%) |
| Metastasis (Lung) | 1 (0.1%) | 1 (0.7%) | 2 (0.2%) |
| Pterygoid-maxillary tumour | 0 (0%) | 2 (1.4%) | 2 (0.2%) |
| Chondrosarcoma | 0 (0%) | 5 (3.6%) | 5 (0.6%) |
| Hemangiopericytoma | 0 (0%) | 1 (0.7%) | 1 (0.1%) |
| Adenocarcinoma (Sinonasal) | 0 (0%) | 5 (3.6%) | 5 (0.6%) |
| Metastasis (Melanoma) | 1 (0.1%) | 1 (0.7%) | 2 (0.2%) |
| Metastasis (Other) | 0 (0%) | 1 (0.9%) | 1 (0.1%) |
| Cavernous haemangioma | 1 (0.1%) | 0 (0%) | 1 (0.1%) |
| Metastasis (Prostate) | 1 (0.1%) | 0 (0%) | 1 (0.1%) |
| **Grand Total** | **726** | **140** | **866** |

Supplementary material 4: Full list of all repair methods per category by approach.

| ***Repair Technique*** | ***Transsphenoidal Approach*** | ***Expanded Endoscopic Endonasal Approach*** |
| --- | --- | --- |
| **Dural Closure** | **0** | **0** |
| Sutures | 0 | 0 |
| Clips | 0 | 0 |
| **Dural Replacement** | **196** | **66** |
| Duragen® | 136 | 43 |
| Fascia Lata | 18 | 12 |
| Lyoplant® | 17 | 0 |
| Duramend® | 7 | 0 |
| Tachosil® | 6 | 6 |
| Tutoplast® | 0 | 6 |
| Durarepair® | 4 | 1 |
| Redura® | 1 | 3 |
| Neuropatch® | 3 | 0 |
| Haemopatch® | 3 | 0 |
| Duraform® | 0 | 2 |
| Duraguard® | 1 | 0 |
| Durapore® | 1 | 0 |
| Ethisorb® | 1 | 0 |
| Fibrillar | 3 | 0 |
| **Tissue Graft** | **221** | **65** |
| Autologous Fat | 189 (abdomen 145, thigh 44) | 45 (abdomen 20, thigh 20, unspecified 5) |
| Autologous Fascia | 27 (Lata 25, unspecified 2) | 36 (Lata 32, temporalis 3, unspecified 1) |
| Autologous Mucosa | 28 (middle turbinate 10, septal 4, sphenoid 13, nasal unspecified 1) | 8 (middle turbinate 1, septal 4, sphenoid 1, nasal unspecified 2) |
| Autologous Bone | 8 (septum 7, vomer 2) | 4 (vomer 2, septum 1, unspecified 1) |
| Autologous Muscle | 4 (thigh 4) | 0 |
| Autologous Cartilage | 1 (septal) | 0 |
| Autologous Periosteum | 0 | 1 (pericranium) |
| **Synthetic Graft** | **204** | **47** |
| Spongostan™ | 181 | 39 |
| Tachosil® | 21 | 5 |
| Gelfoam® | 2 | 1 |
| Collagen sponge | 1 | 0 |
| Gliadel® wafers | 1 | 0 |
| Redura® | 0 | 1 |
| Tutoplast® | 0 | 1 |
| **Pedicled Vascular Flap** | **116** | **90** |
| Nasoseptal flaps | 105 | 87 |
| Middle turbinate flaps | 11 | 2 |
| Mucoperichondrial | 0 | 1 |
| Temporoparietal | 0 | 1 |
| **Tissue Glue** | 489 | 99 |
| Adherus® | 146 | 22 |
| Duraseal® | 137 | 22 |
| Tisseel® | 126 | 32 |
| Evicel® | 43 | 16 |
| Bioglue® | 40 | 7 |
| Stammberger foam® | 2 | 1 |
| Floseal® | 1 | 0 |
| **Haemostatic Agents** | **439** | **93** |
| Surgicel® | 189 | 51 |
| Surgiflo® | 141 | 24 |
| Floseal® | 91 | 13 |
| Fibrillar® | 48 | 3 |
| Gelfoam® | 0 | 5 |
| Lyostypt® | 7 | 2 |
| Haemopatch® | 2 | 2 |
| Thrombin product unspecified | 1 | 0 |
| **Buttress** | **31** | **17** |
| Medpor® polyethylene | 15 | 10 |
| Autologous Bone | 14 (septal 10, sphenoid 4) | 5 (septal 4, unspecified 1) |
| Autologous Cartilage | 1 (unspecified 1) | 2 (septal 1, unspecified 1) |
| Silastic splint | 1 | 0 |
| **Nasal Pack** | **519** | **116** |
| Nasopore® | 369 | 86 |
| Merocel® | 94 | 20 |
| Bismuth-soaked ribbon gauze | 34 | 11 |
| Rapid Rhinos® | 33 | 10 |
| Posisep® | 10 | 5 |
| Stammberger foam® | 9 | 5 |
| Netcell® | 8 | 4 |
| Foley Catheter | 2 | 10 |
| Bactroban®-soaked ribbon gauze | 0 | 7 |
| Sinofoam® | 2 | 0 |
| Parrafin-soaked ribbon gauze | 1 | 0 |
| Unspecified | 3 | 2 |
| **CSF diversion** | **29** | **38** |
| Lumbar drain | 27 | 38 |
| External ventricular drain | 1 | 1 |
| Lumbar puncture | 1 | 0 |
| Ventriculoperitoneal shunt | 1 | 0 |

Supplementary material 5:

Table 5a. Summary of baseline and operative risk factors for CSF rhinorrhoea – incidence and statistical analysis via univariate logistic regression.

|  | Transsphenoidal approach | | Expanded Endonasal Approach | |
| --- | --- | --- | --- | --- |
|  | **CSF Rhinorrhoea rate** | **Univariate Analyses  (OR, CI, p-value)** | **CSF Rhinorrhoea rate** | **Univariate Analyses  (OR, CI, p-value)** |
| **Approach** |  |  |  |  |
| TSA | 28/726 (3.9%) | OR: 0.52, CI: 0.25-1.01, p=0.087 | - | - |
| EEA | - | - | 10/140 (7.1%) | OR: 1.92, CI: 0.91-4.04, p=0.087 |
|  |  |  |  |  |
| **Baseline characteristics** |  |  |  |  |
| Age >65 | 0/172 (0.0%) | - | 3/27 (11.1%) | OR: 1.89, CI: 0.46-7.86, p=0.380 |
| Age <65 | 28/553 (5.1%) | Reference | 7/113 (6.2%) | Reference |
| BMI >30 | 11/210 (5.2%) | OR: 1.67, CI: 0.77-3.59, p=0.192 | 2/28 (7.1%) | OR: 1.00, CI: 0.20-5.00, p=1.000 |
| BMI<30 | 17/516 (3.3%) | Reference | 8/112 (7.1%) | Reference |
| Tumour diameter >1cm | 21/607 (3.5%) | OR: 0.54, CI: 0.23–1.29, p = 0.167 | 10/131 (7.6%) | - |
| Tumour diameter <1cm | 7/119 (6.0%) | Reference | 0/9 (0%) | Reference |
| Primary surgery | 8/98 (8.2%) | **OR: 0.36, CI: 0.15-0.85, p=0.019** | 1/21 (4.8%) | OR: 1.32, CI: 0.15 – 11.33, p=0.800 |
| Revision surgery | 19/573 (3.3%) | Reference | 7/113 (6.2%) | Reference |
| Presence of Otorhinolaryngologist | 9/268 (3.4%) | OR: 0.82, CI: 0.37-1.83, p=0.634 | 8/93 (8.6%) | OR: 2.12, CI: 0.43-10.40, p=0.355 |
| Presence of Neurosurgeon | 25/704 (3.6%) | **OR: 0.22, CI: 0.06-0.79, p=0.021** | 9/137 (6.6%) | OR: 0.14, CI: 0.01-1.70, p=0.123 |
| Intra-operative CSF leak grade |  |  |  |  |
| Grade 0 | 11/512 (2.1%) | Reference | 4/61 (6.6%) | Reference |
| Grade 1 | 3/131 (2.3%) | OR: 1.05, CI: 0.29-3.76, p=0.944 | 1/12 (8.3%) | OR: 1.30, CI: 0.13-12.72, p=0.824 |
| Grade 2 | 9/54 (16.7%) | **OR: 9.35, CI: 3.74-23.37, p < 0.001** | 1/13 (7.7%) | OR: 1.19, CI: 0.12-11.59, p=0.882 |
| Grade 3 | 0/5 (0%) | - | 2/39 (5.6%) | OR: 0.77, CI: 0.13-4.42, p=0.770 |
| Leak present, grade unknown | 5/24 (20.8%) | **OR: 12.3, CI: 3.94-38.43, p < 0.001** | 2/15 (13.3%) | OR: 2.19, CI: 0.36-13.28, p=0.393 |
|  |  |  |  |  |
| **Repair methods** |  |  |  |  |
| Dural closure | - | - | - | - |
| Dural replacement | 11/196 (5.6%) | OR:1.82, CI: 0.83-3.98 p=0.136 | 5/66 (7.6%) | OR: 1.11, CI: 0.31-4.04, p=0.869 |
| Tissue graft | 13/221 (5.9%) | OR: 1.72, CI: 0.82-3.63, p=0.154 | 3/65 (4.6%) | OR: 0.47, CI: 0.12-1.90, p=0.289 |
| Synthetic graft | 7/204 (3.4%) | OR: 0.81, CI: 0.34-1.92, p=0.628 | 6/47 (12.8%) | OR: 3.26, CI: 0.87-12.17 p=0.079 |
| Button Technique | 0/20 (0%) | - | 0/7 (0%) | - |
| Pedicled Flap | 5/116 (4.3%) | OR: 1.17, CI: 0.43-3.17, p=0.756 | 8/90 (8.9%) | - |
| Tissue Glue | 15/474 (3.2%) | OR: 0.62, CI: 0.29-1.34, p=0.226 | 8/114 (7.0%) | OR: 0.83, CI: 0.16-4.18, p=0.821 |
| Haemostatic agent | 18/439 (4.1%) | OR: 1.05, CI: 0.49-2.26, p=0.896 | 5/93 (5.4%) | OR: 0.48, CI: 0.13-1.74, p=0.262 |
| Buttress | 0/31 (0%) | - | 1/17 (5.9%) | OR: 0.78, CI: 0.09-6.61, p=0.789 |
| Gasket sealing | 0/15 (0%) | - | 0/11 (0%) | - |
| Nasal packing | 22/519 (4.2%) | OR: 1.75, CI: 0.65-4.68, p=0.266 | 10/116 (8.6%) | - |
| CSF diversion | 1/29 (3.4%) | OR: 0.87, CI: 0.12-6.64, p=0.896 | 1/38 (2.6%) | OR: 0.28, CI: 0.03-2.28, p =0.234 |

Table 5b. Summary of operative technique and intra-operative CSF leak – incidence and statistical analysis via Fisher’s exact test.

| **Repair methods** | **Intra-operative CSF leak grade during Expanded Endonasal Approach (N = 140)** | | | | | | **Intra-operative CSF leak grade during Transsphenoidal approach (N = 726)** | | | | | |
| --- | --- | --- | --- | --- | --- | --- | --- | --- | --- | --- | --- | --- |
|  | Grade 0 | Grade 1 | Grade 2 | Grade 3 | Leak present, grade unknown | p-value | Grade 0 | Grade 1 | Grade 2 | Grade 3 | Leak present, grade unknown | p-value |
| Dural closure | 0/60 (0%) | 0/12 (0%) | 0/13 (0%) | 0/39 (0%) | 0/13 (0%) | - | 0/505 (0%) | 0/130 (0%) | 0/54 (0%) | 0/5 (0%) | 0/24 (0%) | - |
| Dural replacement | 23/61 (37.7%) | 7/12 (58.3%) | 8/13 (61.5%) | 19/39 (48.7%) | 9/14 (64.2%) | 0.236 | 113/509 (22.2%) | 47/130 (36.2%) | 19/54 (35.2%) | 4/5 (80.0%) | 13/24 (54.2%) | **<0.001** |
| Tissue graft | 18/61 (29.5%0 | 9/12 (75.0%) | 4/13 (30.8%) | 28/39 (71.8%) | 6/15 (40.0%) | **<0.001** | 88/512 (17.2%) | 80/131 (61.1%) | 37/54 (68.5%) | 5/5 (100.0%) | 11/24 (45.8%) | **<0.001** |
| Synthetic graft | 22/61 (36.1%) | 4/13 (33.3%) | 4/13 (30.8%) | 13/39 (33.3%) | 4/15 (26.7%) | 0.985 | 141/512 (27.5%) | 41/131 (31.3%) | 14/54 (25.9%) | 2/5 (40.0%) | 6/24 (25.0%) | 0.835 |
| Button Technique | 2/26 (7.7%) | 0/11 (0%) | 1/7 (14.3%) | 3/30 (10.0%) | 1/5 (20.0%) | 0.560 | 7/171 (4.1%) | 7/89 (7.9%) | 9/54 (16.7%) | 0/5 (0%) | 1/24 (4.2%) | 0.119 |
| Pedicled Flap | 30/60 (50.0%) | 8/12 (66.7%) | 12/13 (92.3%) | 29/38 (76.3%) | 11/11 (100.00%) | **0.001** | 58/475 (12.2%) | 29/121 (24.0%) | 21/47 (44.7%) | 1/4 (25.0%) | 7/24 (29.2%) | **<0.001** |
| Tissue Glue | 41/61 (67.2%) | 12/12 (100%) | 12/13 (92.3%) | 39/39 (100.0%) | 10/13 (76.9%) | **<0.001** | 294/509 (57.8%) | 112/130 (86.2%) | 43/54 (79.6%) | 5/5 (100.0%) | 20/24 (83.3%) | **<0.001** |
| Haemostatic agent | 38/61 (62.3%) | 9/12 (75.0%) | 7/13 (53.9%0 | 27/39 (69.2%) | 12/15 (80.0%) | 0.553 | 320/512 (62.5%) | 73/131 (55.7%) | 26/54 (48.1%) | 1/5 (20.0%) | 19/24 (79.2%0 | **0.013** |
| Buttress | 5/61 (8.2%) | 0/12 (0%) | 1/13 (7.7%) | 9/39 (23.1%) | 2/14 (14.3%0 | 0.147 | 14/508 (2.8%) | 7/129 (5.4%) | 9/54 (16.7%) | 0/5 (0%) | 1/24 (4.2%) | **0.001** |
| Gasket sealing | 3/6 (50.0%) | - | 0/2 (0.0%) | 6/9 (66.7%) | 2/2 (100.0%) | 0.267 | 8/19 (42.1%) | 3/8 (37.5%) | 4/10 (40.0%) | - | 0/1 (0%) | 1.000 |
| Nasal packing | 50/61 (82.0%) | 12/12 (100.0%) | 12/13 (92.3%) | 28/39 (71.8%) | 14/14 (100.0%) | **0.046** | 353/494 (71.5%) | 102/128 (79.7%) | 40/53 (75.5%) | 3/4 (75.0%) | 21/24 (87.5%) | 0.175 |
| CSF diversion | 8/61 (13.1%) | 1/12 (8.3%0 | 4/13 (30.8%) | 16/39 (41.0%) | 9/15 (60.0%) | **<0.001** | 11/512 (2.1%) | 12/131 (9.2%) | 4/54 (7.4%) | 0/5 (0%) | 2/24 (8.3%) | **0.002** |
| **TOTAL** | 61 | 12 | 13 | 39 | 15 |  | 512 | 131 | 54 | 5 | 24 |  |

Supplementary material 6: Summary of visual, endocrine and general outcomes with up to 6 months follow up for transsphenoidal and expanded endonasal cases (if available). SIADH = syndrome of inappropriate anti-diuretic hormone, DI = diabetes insipidus.

|  | Transsphenoidal approach | | Expanded Endonasal Approach | |
| --- | --- | --- | --- | --- |
|  | **Pre-operative** | **Post-operative (if available)** | **Pre-operative** | **Post-operative (if available)** |
| **Visual & Endocrine Outcomes at 6 months** |  |  |  |  |
| Visual deficits (acuity or field) | All deficits: 360/726 (51.7%)  Blind: 9/360 (2.4%) | Worse: 10/239 (4.2%)  Stable: 53/239 (22.2%)  Improved: 176/239 (73.6%) | All deficits: 91/140 (65.0%)  Blind: 3/91 (3.3%) | Worse: 7/56 (12.5%)  Stable: 11/56 (19.6%)  Improved: 38/56 (67.9%) |
| Anterior hypopituitarism requiring steroid replacement | 184/724 (25.4%) | Worse: 131/427 (30.7%)  Stable: 263/427 (61.6%)  Improved: 33/427 (7.7%) | 31/140 (22.1%) | Worse: 22/73 (30.1%)  Stable: 46/73 (63.0%)  Improved: 5/73 (6.8%) |
| Posterior hypopituitarism requiring desmopressin replacement | 28/722 (3.9%) | Worse: 49/421 (11.6%)  Stable: 367/421 (87.2%)  Improved: 5/421 (1.2%) | 8/140 (5.7%) | Worse: 13/74 (17.6%)  Stable: 59/74 (79.7%)  Improved: 2/74 (2.7%) |
| **Postoperative Complications** |  |  |  |  |
| Residual/recurrent disease* | - | 73/726 (3.3%) | - | 10/140 (7.1%) |
| New DI (transient or permanent) | - | 50/726 (6.9%) | - | 15/140 (10.7%) |
| Nasal crusting | - | 45/726 (6.2%) | - | 11/108 (7.9%) |
| SIADH | - | 22/726 (3.0%) | - | 4/140 (2.9%) |
| Hyponatraemia (unspecified) | - | 14/726 (1.9%) | - | 2/140 (1.4%) |
| CNS infection | - | 10/726 (1.4%) | - | 4/140 (2.9%) |
| New focal neurological deficit | - | 12/726 (1.7%) | - | 2/140 (1.4%)  7/140 (5.0%) |
| Epistaxis (requiring surgical intervention) | - | 9/726 (1.2%) | - | 0/140 (0%) |
| All-cause mortality | - | 6/726 (0.8%) | - | 2/140 (1.4%) |
| Hypernatraemia (unspecified) | - | 4/726 (0.6%) | - | 2/140 (1.4%) |
| Seizures | - | 2/726 (0.3%) | - | 0/140 (0%) |
| Major blood vessel injury (e.g. carotids) | - | 3/726 (0.4%) | - | 0/140 (0%) |
| Other | - | 20^a^/726 (2.8%) | - | 5^b^/140 (3.6%) |

^*^ Independent of surgical intention. Includes functional recurrence if functioning tumour.  ^a^ Abdominal wall haematoma x2, psychosis/delirium/confusion x1, sepsis x5, wound breakdown x1, ketosis x1, respiratory infection x4, hyperglycaemia x1,, nasal discharge x1, obstructive hydrocephalus x1, arrythymia x2, otitis media x1

^b^ Lumbar drain leak & intracranial hypotension x1, pulmonary embolus x1, pneumocephalus x1, psychosis/delirium/confusion x2, septal perforation x1,
